# Effectiveness of Intravenous and Nebulized MgSO_4_ in Children with Asthma Exacerbation: A Systematic Review and Meta-Analysis of Clinical Trials

**DOI:** 10.3390/children12081064

**Published:** 2025-08-13

**Authors:** Víctor Hugo Estupiñán Pérez, Freiser Eceomo Cruz Mosquera, Mayerli de la Rosa Caldas, Olmer Alexander Pantoja Rodríguez, Yamil Liscano

**Affiliations:** 1 Grupo de Investigación en Salud Integral (GISI), Department of Health Sciences Faculty, Universidad Santiago de Cali, Cali 760035, Colombia; vestupinan@usc.edu.co (V.H.E.P.); mayerlide@usc.edu.co (M.d.l.R.C.); yamil.liscano00@usc.edu.co (Y.L.); 2 Unidad De Cuidados Intensivos Pediátrica, Hospital Universitario del Valle Evaristo García, Cali 760043, Colombia; oapantojar@hotmail.com

**Keywords:** asthma, child, magnesium sulfate, length of stay, hospitalization, intensive care units

## Abstract

**Background/Objectives:** Moderate or severe asthma exacerbations may require pharmacological interventions in addition to standard treatment. In this context, magnesium sulfate has been proposed as a second-line therapeutic option, owing to its physiological effects on bronchial smooth muscle. Therefore, the objective of this meta-analysis is to determine the effectiveness of intravenous or nebulized magnesium sulfate in patients with a moderate-to-severe asthmatic crisis. **Methods:** This systematic review and meta-analysis included randomized controlled trials published between 1990 and 2024, using the PubMed, Scopus, Science Direct, Web of Science, LILACS, Cochrane Library, Springer, and Scielo databases. The risk of bias was assessed using the RoB 2 tool, the quality of evidence with the Jadad scale, and the certainty of the evidence per outcome was evaluated following the GRADE guidelines. The meta-analysis was developed using the statistical software Jamovi 2.3.28^®^ and RevMan 5.4^®^. **Results:** Fourteen studies with a total of 2242 patients with a moderate-to-severe asthmatic crisis were included. Of these, ten studies evaluated the severity score, eight evaluated hospitalization, five evaluated the length of the hospital stay, and three evaluated intensive care unit admission. The meta-analysis demonstrates that the use of magnesium sulfate is associated with a statistically significant reduction in the risk of hospitalization (RR: 0.79, 95% CI: from 0.67 to 0.94, *p* = 0.02). However, no effects were observed on the severity score (SMD: −0.37, 95% CI: from −0.92 to 0.17, *p* = 0.16), the length of the hospital stay (SMD: −0.75, 95% CI: from −1.90 to 0.40, *p* = 0.14), or admission to intensive care units (RR: 0.62, 95% CI: from 0.28 to 1.36, *p* = 0.23). Subgroup and sensitivity analyses did not yield significant findings or produce any modification of the effect. **Conclusions:** Magnesium sulfate reduces hospitalizations in moderate-to-severe pediatric asthma, although it does not improve other relevant clinical outcomes.

## 1. Introduction

Asthma is one of the most prevalent chronic diseases in childhood, with a considerable impact on quality of life, school performance, and the use of healthcare services [1,2,3]. Its burden has increased in recent decades, especially in countries with accelerated processes of urbanization and environmental change [4,5]. Although prevalence varies by region, it is estimated to affect 20% of the pediatric population, and at least 3% of children present frequent symptoms that lead to frequent emergency department visits and hospitalizations each year [6,7].

In the pediatric context, severe asthma is characterized by the persistence of respiratory symptoms despite the appropriate use of short-acting bronchodilators and systemic corticosteroids [8,9,10]. Airflow obstruction, which is generally reversible, can reach a clinically significant intensity during acute exacerbations, compromising oxygenation and creating a life-threatening risk in the absence of timely intervention [11,12]. In this situation, the British Thoracic Society (BTS) and international guidelines, such as the Global Initiative for Asthma (GINA), have recommended the use of magnesium sulfate (MgSO_4_), administered intravenously (IV) or nebulized, as an adjuvant treatment in children with moderate-to-severe exacerbations and who do not respond to conventional therapy [13,14,15].

Magnesium sulfate exerts bronchodilator and anti-inflammatory effects through multiple physiological mechanisms. Among them, the competitive inhibition of calcium influx into bronchial smooth muscle, which reduces muscle contraction, stands out, as well as the modulation of cholinergic neuromuscular transmission and the stabilization of mast cells [16]. These physiological effects contribute to the relaxation of the airway smooth muscle and decreases in bronchial edema and inflammation [17]. Despite magnesium sulfate’s growing clinical use, the available evidence in the pediatric population has significant limitations, including heterogeneity in study designs, differences in dosages, routes of administration, outcomes assessed, and comparators used [15,18,19].

While some clinical trials have shown the benefits of intravenous MgSO_4_ as an adjuvant treatment in acute exacerbations, including improvement in clinical severity scores and a reduction in the need for hospitalization, its overall effectiveness remains a matter of controversy [20,21,22]. The intravenous route, although potentially more effective in pharmacodynamic terms, is associated with adverse effects, such as pain at the puncture site, hypotension, nausea, vomiting, and mild systemic reactions, which warrant close clinical monitoring during its administration [23]. For its part, the nebulized route has been proposed as a less invasive alternative, with a lower rate of adverse events by limiting systemic absorption [14,24]. However, various studies have suggested that its bronchodilator effect may be less potent compared to that of the IV route [25].

Given the inconsistency of the findings and the lack of a clinical consensus on the routine use of magnesium sulfate in pediatric patients with acute asthma, the present article aims to conduct a systematic review and a meta-analysis of the current scientific literature to evaluate the efficacy and safety of MgSO_4_, by both intravenous and nebulized routes, as an adjuvant treatment in the management of moderate-to-severe asthma exacerbations in children.

## 2. Materials and Methods

### 2.1. Study Protocol

This investigation was conducted as a systematic review with a meta-analysis, following the established guidelines in the PRISMA statement and the *Cochrane Collaboration Handbook*. To ensure methodological transparency, the research protocol was registered with PROSPERO (available at https://www.crd.york.ac.uk/prospero/) during 14 March 2025, under registration number CRD420251010206. The formulation of the primary research question was structured using the PICO framework (population, intervention, comparison, and outcomes).

### 2.2. Research Question

In pediatric patients with acute asthma (P), does the administration of magnesium sulfate as a monotherapy or an adjuvant treatment (I), compared to standard care or a placebo (C), demonstrate efficacy in improving severity scores and reducing hospitalizations, the hospital length of stay, intensive care unit admissions, mortality, and adverse events (O)?

### 2.3. Eligibility Criteria

#### 2.3.1. The Eligibility Criteria for Inclusion in the Review

The eligibility criteria for inclusion in the review were as follows:

- Randomized controlled clinical trials, regardless of the follow-up duration and specific study design (crossover, parallel);
- Studies with multiple study groups were included when the arm assigned to magnesium sulfate treatment alone or as an adjuvant therapy could be identified independently;
- Scientific publications available between January 1990 and December 2024;
- Articles available in Spanish, Portuguese, or English languages;
- Research conducted in pediatric populations with acute asthma, evaluating the effectiveness of magnesium sulfate as a monotherapy or an adjuvant therapy, administered intravenously or via nebulization, at any dosage, administration timing, or therapeutic regimen, compared with placebo or standard treatment proposed by the trial authors;
- Studies that assessed at least one of the following outcomes: severity score, hospitalization, hospital length of stay, intensive care unit admission, mortality, and adverse events.

#### 2.3.2. Exclusion Criteria

The exclusion criteria established for the review were as follows:

- Preprint articles;
- Conference abstracts;
- Letters to editors;
- Studies unavailable in an accessible format;
- Research utilizing the same patient cohort as other clinical trials conducted for identical purposes;
- Studies that failed to specify the exact dosage administered to the patients;
- Animal model studies.

### 2.4. Data Sources and Search Strategy

Scientific evidence was sought from the following databases: LILACS, Web of Science, Cochrane Clinical Trials, Scopus, Scielo, Springer, Science Direct, and PubMed. Where applicable, language filters (Spanish, Portuguese, and English) and the predefined date range were utilized. Three independent investigators were responsible for the formulation and execution of the search strategy (V.H.E.P., M.R.C., and F.C.M.) between March and April 2025, using the following syntax:

(Asthma OR “acute asthma” OR “asthma exacerbation”) AND (Child OR pediatric) AND (Magnesium Sulfate OR “MgSO4”) AND (“Nebulizers and Vaporizers” OR nebulized OR inhaled OR aerosolized) OR (“Infusions, Intravenous” OR intravenous OR IV) AND (Randomized Controlled Trial OR Clinical Trial).

This strategy was adapted according to each database’s specific requirements. Additionally, a manual review of reference lists from relevant studies was performed to identify research not captured by initial database searches, and a manual web search was conducted for supplementary studies. When additional information about a study was required, records from ClinicalTrials.gov (https://clinicaltrials.gov/, accessed in 3 April 2025) or the designated repository for clinical trial protocols were consulted. Data storage and management were facilitated using Rayyan–Intelligent Systematic Review version AI (https://www.rayyan.ai/, accessed in 7 April 2025).

### 2.5. Study Selection and Data Extraction

Two independent reviewers initially screened the studies to identify those potentially eligible for inclusion (V.H.E.P and O.A.P.R.) through the evaluation of titles, abstracts, and, subsequently, full-text articles. In cases where complete study access was unavailable, the corresponding authors were contacted. Discrepancies in study inclusion were resolved through discussion and consensus. When initial agreement was not reached, a third reviewer (Y.L.) provided definitive decision making. Cohen’s kappa coefficient was utilized to measure inter-rater agreement and assess consistency during the study selection.

Data extraction from selected investigations was conducted by two reviewers (Y.L. and V.H.E.P.), collecting key study details (first author, publication year, and country), participant characteristics (number of subjects per group, age, asthma crisis severity, and gender), intervention specifications (dosage, administration route, administration timing, and adjuvant therapy), and evaluating outcomes. The integrity and accuracy of the extracted data were later verified by two additional reviewers (Y.L. and F.C.M.).

#### Outcomes Evaluated in the Review

The outcomes considered in this systematic review and meta-analysis were improvements in asthma severity score, hospitalization rate, hospital length of stay, intensive care unit admission, mortality, and adverse events. The primary outcome was variation in the severity score, as it constitutes a direct clinical marker of therapeutic response in the context of acute exacerbation. The selection of this outcome was based on its sensitivity to detect early clinical changes and its widespread use in pediatric acute care settings, where severity scales are standardized tools for evaluating treatment efficacy. The remaining outcomes were considered as secondary, as they reflect subsequent clinical events.

### 2.6. Risk-of-Bias Assessment

Risk of bias in the included clinical trials was assessed independently by reviewers F.C.M. and M.R.C., employing a standard tool designed for this study type. Collected assessment information was recorded in Review Manager version 5.4^®^. The evaluation criteria encompassed the (a) generation of the randomization sequence, (b) concealment of the group assignment, (c) blinding of the participants and study staff, (d) blinding in the evaluation of outcomes, (e) management of incomplete outcome data, and (f) risk of selective outcome reporting. Each trial was assessed and classified as presenting a low, unclear, or high risk of bias according to predefined methodological standards. Any discrepancies between reviewers were addressed and resolved through consensus discussions [26].

### 2.7. Assessment of the Quality of Evidence

The internal validity of the clinical trials included in the meta-analysis was systematically appraised through the application of the Jadad quality assessment tool (from 0 to 5 points, with higher scores indicating better quality), based on the following criteria: (a) Participants were assigned using a randomized design; (b) the intervention was administered under double-blind conditions; (c) withdrawals and losses to follow-up were described; (d) the randomization method was adequately reported; (e) selection criteria were clearly described. Each criterion was scored as 1 if adequately reported or 0 if absent or insufficiently described. Trials scoring 0–2 were considered as low quality, while those scoring ≥3 were considered to have acceptable methodological quality. Although no study was excluded based on this assessment, the quality scores were considered in the interpretation of the results [27].

### 2.8. Statistical Analysis

RevMan 5.4^®^ software (accessed in 27 April 2025) was utilized for meta-analysis. Effect sizes and their 95% confidence intervals were calculated. Meta-analysis was performed when at least two studies evaluated any of the following outcomes: asthma severity score, hospitalization, hospital length of stay, and intensive care unit admission. For quantitative outcomes, means and standard deviations were extracted from primary studies. When results were reported as medians and interquartile ranges, appropriate statistical conversions were applied. For outcomes assessed at multiple time points, the final recorded value for each group was used in the analysis. Relative risk was employed for dichotomous outcomes, while for quantitative outcomes, the mean difference (MD) or standardized mean difference (SMD) was used when measurement units or scoring scales varied. Mortality and adverse effects were not included in the meta-analysis due to their number or reporting variability; instead, qualitative synthesis was performed to present findings.

Subgroup analyses were performed according to age group, route of administration, dose, timing of drug administration, and asthma severity. These analyses were defined a priori in the study protocol. Age was categorized into preschool, school-age, and adolescent groups; the route of administration was classified as nebulized or intravenous; the timing of the administration was defined as less than or greater than 12 h from hospital admission; and asthma severity was categorized as moderate or severe. Subgroup analysis was performed when possible, as in several studies, the data were presented in a combined manner or with incomplete reporting, which prevented adequate discrimination of results by category. An additional post hoc subgroup analysis was proposed according to the timing of the magnesium sulfate initiation (from the beginning or after management with SABA, SAMA, or oral or systemic corticosteroids) and the existence or absence of co-interventions. Likewise, sensitivity analysis was performed, when necessary, through the exclusion of individual studies and those that, according to assessment using the Jadad scale, did not present double blinding or showed deficiencies in the description of the randomization process.

For studies with zero events in one or both comparison groups, a continuity correction of adding 0.5 to each cell of the 2 × 2 contingency table was applied. This correction facilitated the calculation of relative risks and their confidence intervals, preventing undefined estimates and ensuring the inclusion of studies with infrequent events in the meta-analysis. Statistical heterogeneity was assessed using the I^2^ statistic, with values reaching or exceeding 50% considered as high. A fixed-effect model was applied when I^2^ was below 50%, whereas a random-effect model was used when this threshold was surpassed. In both scenarios, the inverse variance method was employed. A statistical significance threshold of *p* < 0.05 was established. Finally, publication bias was explored through funnel plots and Egger’s test, using Jamovi^®^ software, version 2.3 (accessed in 27 April 2025). The certainty of evidence was assessed using the GRADE approach, which examines five key aspects: risk of bias, inconsistency, imprecision of estimates, indirectness, and potential publication bias. All the meta-analysis results were analyzed and presented in a summary of findings (SoF) table. Based on methodological evaluation and coherence of findings, evidence certainty was categorized as high, moderate, low, or very low.

## 3. Results

### 3.1. Studies Identified for the Review

**Figure 1:**
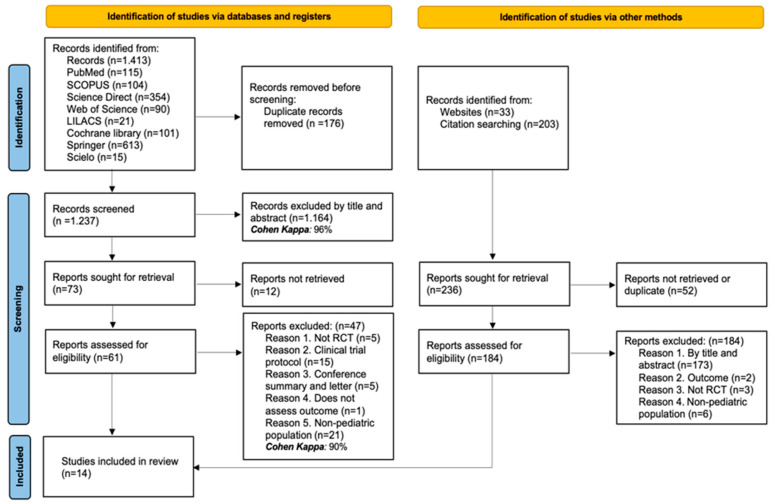
PRISMA flow diagram with the search and study selection.

The initial search yielded 1413 records across the targeted databases. After removing 176 duplicate records, 1237 were kept for screening by title and abstract. Of these, 1164 were excluded (Cohen’s kappa coefficient: 96%), leaving 73 reports for retrieval. Twelve of them could not be retrieved, and sixty-one full-text articles were assessed. Forty-seven articles were removed based on the criteria outlined below: five were not randomized clinical trials, fifteen were study protocols, five were conference abstracts or letters to the editor, one did not assess the outcome of interest, and twenty-one included a non-pediatric population. As a result, 14 RCTs were included in the systematic review (Cohen’s kappa coefficient: 90%). Additionally, 236 records were identified through other methods (website searches and citation reviews). Fifty-two records were duplicates or could not be retrieved. The remaining one hundred eighty-four were assessed in full-text, all of which were excluded: one hundred seventy-three by title and abstract, two for not addressing the defined outcomes, three were not RCTs, and six included a non-pediatric population. In total, 14 studies were included in the systematic review [28,29,30,31,32,33,34,35,36,37,38,39,40,41]. See the PRISMA flow diagram (Figure 1) for more details.

### 3.2. Characteristics of the Studies Included in the Review

The included studies are randomized clinical trials conducted between 1996 and 2024 in various geographical contexts spanning Asia, Europe, the Americas, and the Middle East. This suggests a broad clinical applicability of the use of magnesium sulfate in acute pediatric asthma exacerbations, although it also introduces unavoidable population and healthcare heterogeneity. Regarding the sample size, substantial differences were observed, with studies including from 20 [39] to 816 participants [32].

The characteristics of the population show a homogeneous trend toward the inclusion of pediatric patients, with age ranges predominantly between 2 and 12 years, although some studies extended the upper limit to 17 or 18 years [32,37], and others included children aged 1 year and over [29,40]. In all the cases, the population consisted of patients diagnosed with asthma and a moderate or severe acute exacerbation. In this regard, most studies used objective clinical criteria to define severity, with the recurrent use of validated scales, such as the Pediatric Respiratory Assessment Measure (PRAM), being prominent, with cutoff points generally set at values equal to or greater than 4 [28,30,34]. Some studies supplemented or replaced these clinical scales with functional parameters, such as a peak expiratory flow rate of below 60–70% [39,41].

The inclusion criteria share a common basis centered on the diagnosis of asthma in an acute phase but with relevant nuances between studies. While some trials were limited to severe exacerbation episodes with no response to the initial standard treatment [28,39], others incorporated as a criterion the existence of multiple previous exacerbations with a positive response to bronchodilator use or refractoriness to three doses of nebulized salbutamol [30]. In general, these criteria reinforce the rationale for using MgSO_4_ as a second-line therapeutic strategy in patients with a poor initial response. The exclusion criteria were relatively conservative and homogeneous, prioritizing the pharmacological and methodological safety of the studies. Patients with non-asthmatic chronic respiratory diseases (such as cystic fibrosis or bronchopulmonary dysplasia), cardiac or renal pathologies, and a history of hypersensitivity to magnesium sulfate were systematically excluded. Additionally, several studies excluded patients with a fever higher than 38–38.5 °C [38,40], recent use of theophylline or MgSO_4_ [31,41], or severe clinical conditions requiring immediate intubation or intensive management [28,34]. See the details in Table 1.

### 3.3. Characteristics of the Population and the Intervention

In total, 36% of the studies included patients with a severe asthma exacerbation, 14% with a moderate asthmatic crisis, and the remaining percentage were classified as moderate-to-severe crises. On the other hand, there was a homogeneous distribution in terms of the frequency of the route used for administering magnesium sulfate (50% nebulized and 50% intravenous). In the studies with nebulized administration, most employed three consecutive doses, with a total treatment duration of approximately 60 min [28,29,33,34,35]. The most common dose was 150 mg, used in at least four studies [29,30,33,35]. Other doses included 600 mg [32], 750 mg [28], and 800 mg [34]. In general, the MgSO_4_ was diluted in normal saline solution, with volumes ranging between 1.5 and 15 mL, or in sterile water [30]. In studies that used the intravenous route, administration was performed as a single dose, with infusion times between 20 and 35 min. Doses ranged from 25 mg/kg [38,41] to 100 mg/kg [40]. The most commonly used intermediate doses were 50 mg/kg [31,36] and 40 mg/kg [39].

Dilution volumes ranged from 30 to 100 mL, using saline solution or 5% dextrose. Regarding comparators, eight studies used nebulized saline solution as a placebo [28,32,34,35,37,38,40,41]. Other studies used active comparators, such as salbutamol [29,36], ipratropium bromide with fenoterol [30], and aminophylline [31]. See the details in Table 2. The data related to the timing of the magnesium sulfate initiation, the time between the initial management and intervention administration, and the presence of co-interventions are presented in detail in Table S1 of the Supplementary Materials.

### 3.4. Results of the Risk-of-Bias Assessment

**Figure 2:**
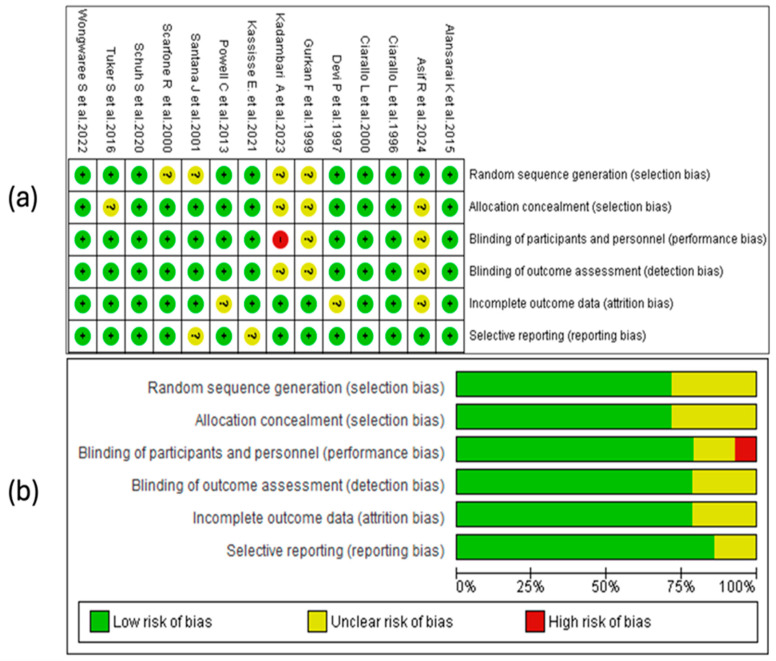
Risk-of-bias assessment for the studies included in this review. (**a**) The symbols used indicate the level of bias risk: “+” corresponds to low risk, “?” denotes unclear risk, and “–“ indicates high risk. These are color-coded as green (low risk), yellow (unclear), and red (high risk). (**b**) The second panel summarizes the distribution of risk of the bias across all the studies, presenting the percentage of evaluations for each bias domain [28,29,30,31,32,33,34,35,36,37,38,39,40,41].

The risk-of-bias analysis of the included studies was conducted considering various methodological domains, as illustrated in Figure 2. The assessment was performed using the RevMan 5.4^®^ tool (accessed in 29 April 2025), allowing the identification of strengths and limitations in the design and the conduct of the analyzed clinical trials. See Figure 2.

#### 3.4.1. Random Sequence Generation

A substantial proportion of the studies considered in this review (10/14) presented a low risk of bias regarding the generation of the random sequence. This suggests that, in general, the randomization methods were adequate and sufficiently described, which strengthens the internal validity of the trials. No studies with high risk in this domain were identified, although a smaller proportion presented an unclear risk due to the lack of detailed information on the procedures used.

#### 3.4.2. Allocation Concealment

Seventy-one percent of the included studies adequately reported allocation concealment, indicating a low risk of bias in this domain. In the remaining 29% [27,28,32,38], the risk was classified as unclear due to an insufficient description of the methods used. No study presented a high risk of bias.

#### 3.4.3. Blinding of Personnel and Participants

Eleven studies (79%) presented a low risk of bias in this domain, demonstrating adequate implementation of blinding. Two trials (14%) showed an unclear risk due to a lack of methodological details, while one (7%), conducted by Devi, P. et al. [39], was classified as high risk, indicating a possible exposure of the groups to the knowledge of the intervention.

#### 3.4.4. Blinding of the Outcome Assessment

In this domain, eleven of the fourteen included studies were assessed as low risk, while the remaining three presented an unclear risk, with no studies identified as high risk. The proper implementation of blinding in most trials helps to reduce the possibility of detection bias, which is particularly relevant for clinical outcomes subject to interpretation, such as the severity score. The studies with an unclear assessment did not provide sufficient information on the procedures used to ensure the blinding of the assessors, which limits the ability to judge the reliability of the obtained results.

#### 3.4.5. Incomplete Outcome Data

The handling of missing data was adequate in most trials, with 11 studies classified as having a low risk of bias. In three studies, the risk was unclear due to a lack of information on the procedures used. The absence of high-risk studies indicates adequate overall management of attrition, although some methodological uncertainty persists in a subgroup of studies.

#### 3.4.6. Selective Reporting

In the domain of selective reporting bias, 12 of the 14 included studies were assessed as low risk, indicating that no evident discrepancies were identified between the expected and presented results in the publications. In two studies [31,36], the risk was classified as unclear due to a lack of access to the registered protocol or insufficient information to verify if all the planned outcomes were reported. See Figure 2a.

#### 3.4.7. Summary of Risk of Bias

The risk-of-bias assessment revealed that approximately 75% of the studies had low risks in random sequence generation, blinding of participants and personnel, outcome assessment, and incomplete data. Allocation concealment showed a low risk in about 70%. Selective reporting bias was the best-rated domain, with over 79% of the studies at low risk. Only a small percentage presented a high risk in any of the domains, mainly in the blinding of personnel. These results suggest adequate overall methodological quality, with opportunities for improvement in the implementation and reporting of allocation concealment and blinding. See Figure 2b.

### 3.5. Qualitative Synthesis of the Scientific Evidence

#### 3.5.1. Adverse Events

Ten of the fourteen included studies evaluated adverse events as a primary or a secondary outcome. Most of the included studies reported a favorable safety profile for magnesium sulfate in the treatment of acute asthma in the pediatric population. Adverse events, when they occurred, were predominantly mild and self-limiting. Some studies reported transient symptoms, such as nasal stinging, nausea, vomiting, or epigastric pain [28,30,40], without major clinical implications. In studies like those by Alansarai, K. et al. [34] and Powell, C. et al. [35], the more severe events were mainly related to the natural course of asthma and not to the study intervention. Other works [31,36,39,41] did not report clinically significant adverse events or hemodynamic alterations attributable to the administration of MgSO_4_. Overall, the available data suggest that the use of magnesium sulfate in this context is safe, provided it is administered under controlled protocols.

#### 3.5.2. Mortality

None of the studies included in this review evaluated mortality as a primary or a secondary outcome. This absence prevents drawing any inference regarding the impact of the intervention on patient survival. The lack of data on mortality limits the comprehensive assessment of clinical effects.

### 3.6. Meta-Analysis

The meta-analysis was conducted for the following outcomes: the severity score, hospitalization, length of the hospital stay, and admission to the intensive care unit. A total of 14 studies were considered [28,29,30,31,32,33,34,35,36,37,38,39,40,41], with a population of 2242 patients.

#### 3.6.1. Results of the Evidence Quality Assessment

The quality of the included studies was assessed using the Jadad scale, which evaluates randomization, masking, reporting of withdrawals, description of the random allocation procedure, and clarity of inclusion criteria. Of the 14 studies analyzed, the majority (n = 8) obtained the maximum score of 5, reflecting high methodological quality and low risk of bias. Three studies [35,36,37] achieved a score of 4, while the studies by Asif et al. [28], Gurkan et al. [39], and Kadambari et al. [29] had scores of 3, mainly due to a lack of double-blinding or an insufficient description of allocation methods. Overall, the evidence is characterized by a predominance of clinical trials with adequate methodological rigor. See Table 3.

#### 3.6.2. Severity Score

**Figure 3:**
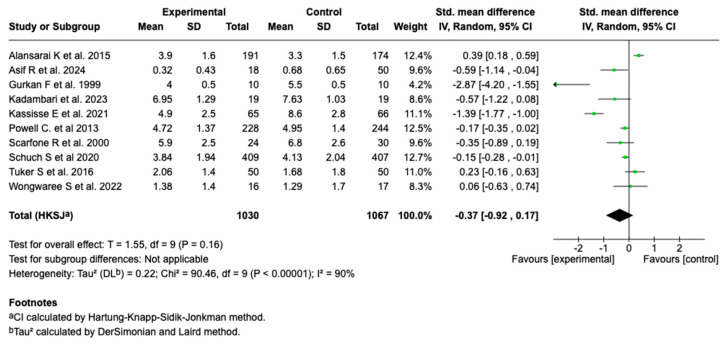
Forest plot of the effect of magnesium sulfate on the severity score of patients with an asthmatic crisis [27,28,29,30,31,32,33,34,36,38].

Ten of the fourteen studies included in the meta-analysis (n = 10; N = 2097) evaluated the effect of intravenous magnesium sulfate on the asthma severity score. The meta-analysis showed a standardized mean difference (SMD) of −0.37 (95% CI: from −0.92 to 0.17; *p* = 0.16), with no evidence of a significant effect compared to the control group (see Figure 3).

**Figure 4:**
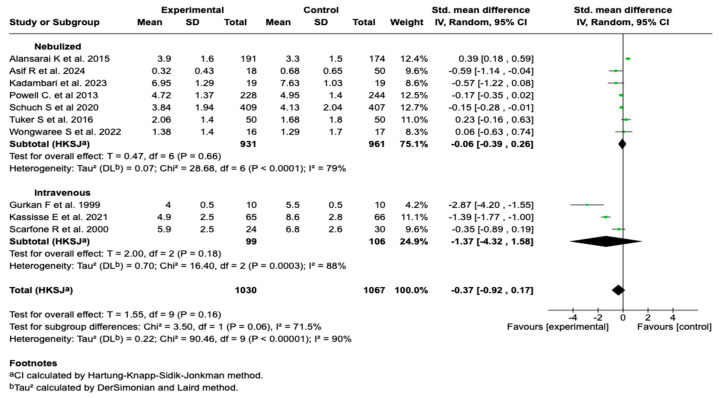
Subgroup analysis by route of administration of magnesium sulfate on the severity score in patients with an asthmatic crisis [28,29,30,31,32,33,34,35,37,39].

Due to the high degree of heterogeneity observed in the overall analysis (I^2^ = 90%), a subgroup analysis was performed according to the route of administration. No statistically significant differences were found between the subgroups (*p* = 0.06), although a trend toward a greater effect was evident in studies using the intravenous route. See Figure 4. Subgroup analysis according to the timing of the magnesium sulfate administration and the presence of co-interventions did not show that these variables were significantly associated with a reduction in the clinical severity score. These findings suggest that neither the timing of the intervention initiation nor the concomitant administration of other standard treatments substantially modified the effect of the magnesium sulfate on this outcome. See Figures S1 and S2 in the Supplementary Materials.

#### 3.6.3. Hospitalization

**Figure 5:**
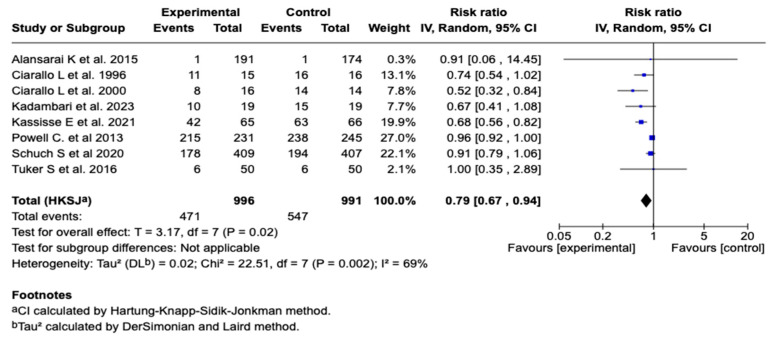
Forest plot of the effect of magnesium sulfate on hospitalization in patients with an asthmatic crisis [29,31,32,33,34,35,38,41].

Eight studies, with one thousand nine hundred eighty-seven patients, evaluated the association between the use of magnesium sulfate and the need for hospitalization in patients with a moderate or a severe asthmatic crisis. The meta-analysis showed a statistically significant reduction in the risk of hospitalization in the group that received magnesium sulfate compared to the control group (relative risk: 0.79; 95% CI: from 0.67 to 0.94; *p* = 0.02), suggesting a protective effect. See Figure 5. Subgroup analysis according to the route of administration showed a significant reduction in the hospitalization risk with the intravenous route (RR = 0.67; 95% CI: 0.50–0.90) but not with the nebulized route (RR = 0.95; 95% CI: 0.91–1.00). The difference between the subgroups was statistically significant (*p* < 0.00001). See Figure S3. On the other hand, subgroup analysis showed that magnesium sulfate administration without concomitant co-interventions was associated with a greater reduction in hospitalization risk (RR = 0.65; 95% CI: 0.54–0.77) compared to studies that included co-interventions (RR = 0.92; 95% CI: 0.86–0.99). The difference between the subgroups was statistically significant (*p* = 0.0002), suggesting a possible modulatory effect of co-interventions on the magnesium sulfate’s efficacy. See Figure S4.

#### 3.6.4. Length of Hospital Stay

**Figure 6:**
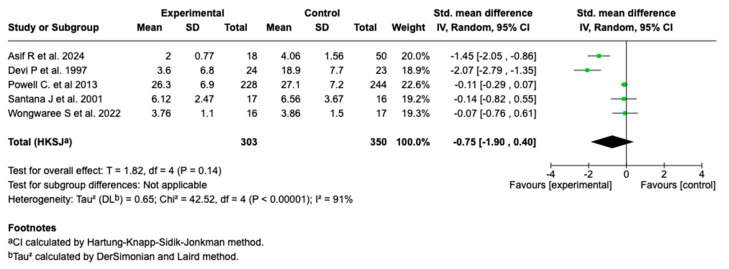
Forest plot of the effect of magnesium sulfate on the length of hospital stay in patients with an asthmatic crisis [28,30,35,36,40].

Five studies (n = 653) evaluated the effect of magnesium sulfate on the length of hospital stay in patients with a moderate or a severe asthmatic crisis. The main analysis showed no statistically significant effect (SMD: −0.75; 95% CI: from −1.90 to 0.40; *p* = 0.14). See Figure 6.

**Figure 7:**
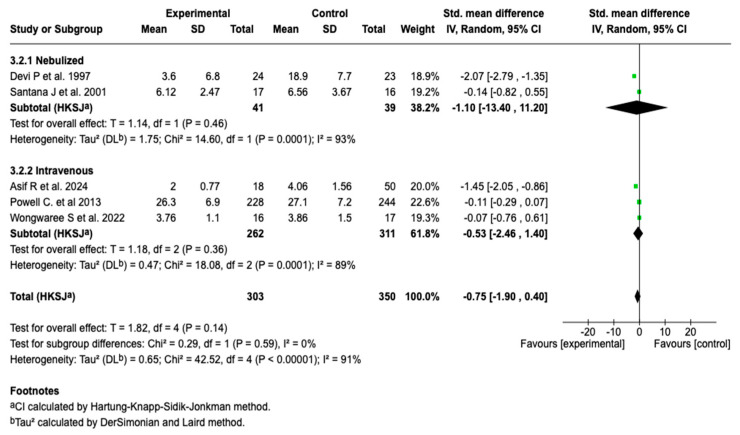
Subgroup analysis by route of administration for the effect of magnesium sulfate on the length of hospital stay in patients with an asthmatic crisis [28,30,35,36,40].

Due to the high degree of heterogeneity (91%), a subgroup analysis was performed according to the route of administration. This analysis showed no statistically significant differences in the length of hospital stay between the nebulized and intravenous forms (*p* = 0.59), suggesting that the route of administration is not differentially associated with this outcome (see Figure 7). Despite this stratification, the heterogeneity degree remained high in both subgroups (I^2^ = 93% and 89%, respectively), which limits the interpretation of the results.

#### 3.6.5. Intensive Care Unit Admission

**Figure 8:**
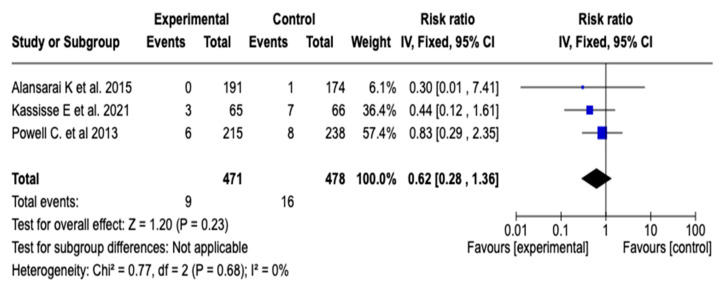
Forest plot of the effect of magnesium sulfate on the risk of admission to the intensive care unit for patients with an asthmatic crisis [31,34,35].

The effect of magnesium sulfate on the need for admission to intensive care was evaluated in three studies (n = 949). The meta-analysis did not show a statistically significant reduction in the risk of ICU admission (RR: 0.62; 95% CI: from 0.28 to 1.36; *p* = 0.23), with no heterogeneity among the studies (I^2^ = 0%). See Figure 8. Given this result, a subgroup analysis was not considered necessary.

#### 3.6.6. Sensitivity Analysis

None of the sensitivity analyses performed, in which studies that presented an inadequate description of the randomization process or that were not double-blind according to the Jadad scale were excluded, substantially modified the direction or magnitude of the effect for any of the evaluated outcomes. These findings support the robustness of the meta-analysis results in the face of methodological limitations of the included studies. (See Figures S5–S8 of the Supplementary Materials).

#### 3.6.7. Results of the Publication-Bias Assessment

**Figure 9:**
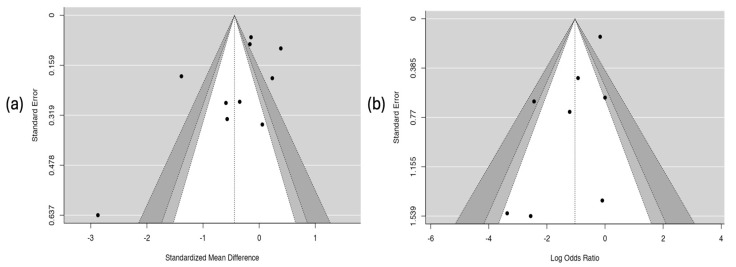
Risk of publication bias. Part (**a**) hospitalization. Part (**b**) severity score of the asthmatic crisis.

The analysis of publication bias is presented in Figure 9. The funnel plot for the hospitalization outcome (Figure 9a) shows clear asymmetry in the distribution of studies, which suggests the possible presence of publication bias. This observation was corroborated by Egger’s test, which showed a statistically significant relationship (coefficient = –2.58; *p* = 0.010), indicating that studies with smaller or non-significant effects may not have been published or included. In contrast, the plot for the asthma crisis severity score (Figure 9b) showed a symmetrical distribution of studies and a non-significant result in Egger’s test (coefficient = –0.357; *p* = 0.275), which suggests a low probability of publication bias for this outcome.

#### 3.6.8. Results of the GRADE Certainty Evidence Assessment

The certainty of the evidence, assessed using the GRADE approach, was moderate for the outcome of ICU admission, low for hospitalization, and very low for the severity score and length of hospital stay. The downgrade in certainty was mainly due to the risk of bias in the included studies, inconsistency among the results (especially in continuous outcomes, with I2 values exceeding 90%), and the imprecision of the estimated effects, with wide confidence intervals that included both benefit and no effect. These findings limit confidence in the results and underscore the need for randomized clinical trials with a low risk of bias and greater statistical power. See Table 4 and Table S2 of the Supplementary Materials.

## 4. Discussion

### 4.1. Main Findings

This review and meta-analysis was conducted to determine the efficacy of magnesium sulfate in the care of pediatric patients with a moderate or a severe asthmatic crisis. Fourteen RCTs were included, with more than two thousand two hundred forty-two participants recruited in different clinical settings. The meta-analysis demonstrated a statistically significant reduction in the risk of hospitalization among patients treated with magnesium sulfate (RR: 0.79; 95% CI: from 0.67 to 0.94; *p* = 0.02), which suggests a potential protective effect in the context of acute exacerbations. However, other clinically relevant outcomes did not reach statistical significance. The administration of magnesium sulfate was not associated with a significant improvement in the crisis severity score (SMD: −0.37; 95% CI: from −0.92 to 0.17; *p* = 0.16) nor with a significant reduction in the length of hospital stay (SMD: −0.75; 95% CI: from −1.90 to 0.40; *p* = 0.14). Likewise, no significant decrease in the need for admission to intensive care units was observed. These findings suggest that while magnesium sulfate might help to prevent the initial hospitalization, its impact on clinical progression and the required treatment intensity appears to be limited or variable. The subgroup analyses, conducted according to the route of administration (intravenous vs. nebulized), did not reveal statistically significant differences between the subgroups in any of the evaluated outcomes. Despite observing isolated trends, these were neither consistent nor clinically conclusive. Furthermore, several outcomes showed high levels of heterogeneity (e.g., I^2^ > 90% in the clinical severity score and length of hospital stay), which limits the precision of the overall estimates and underscores the need to standardize definitions and protocols in future studies. From a safety perspective, the findings of the qualitative synthesis indicated that magnesium sulfate was well tolerated in most studies, with adverse events reported as mild, transient, and without relevant clinical consequences. No serious effects directly related to the drug were documented, which support its safety profile when administered under controlled conditions. Finally, none of the included studies evaluated mortality as an outcome, which represents a significant limitation when comprehensively assessing the clinical impacts of magnesium sulfate. Although mortality in the context of pediatric asthma is infrequent, its exclusion prevents any inference about the treatment’s effect in terms of survival.

### 4.2. Comparison with Previous Studies

The findings of this meta-analysis partially support the efficacy of magnesium sulfate in treating moderate or severe asthmatic crises in the pediatric population. In agreement with previous studies, a statistically significant reduction in the risk of hospitalization was observed in patients treated with magnesium sulfate (RR: 0.79; 95% CI: from 0.67 to 0.94). This result is consistent with the findings reported by Cheuk, D. et al. [42] (OR: 0.29; IC 95%: from 0.14 to 0.59); Su, Z. et al. [43] (RR: 0.55; IC 95%: from 0.31 to 0.95); Mohammed, S. et al. [20] (RR: 0.70; IC 95%: from 0,54 to 0.90); and Shan et al. [23] (RR: 0.70; IC 95%: from 0.54 to 0.91), who also showed a significant protective effect of intravenous magnesium sulfate in reducing hospital admission. The consistency in the direction of this effect reinforces the robustness of the observed benefit, especially in contexts where the intervention is used as a therapy complementary to standard treatment (β_2_-agonists and corticosteroids). A relevant observation from the findings is the reduction in the hospitalization rate in some studies, without a consistent association with other clinical outcomes, such as improvement in the severity score, the length of hospital stay, or ICU admission. This discrepancy may be related to differences in hospitalization criteria between centers, variability in clinical decision making, and uncontrollable factors, such as co-interventions, the baseline severity, or the timing of the treatment administration.

However, similar to our analysis, several studies have not reported significant differences in other clinically relevant outcomes. Kumar, J. et al. [44] and Ling, Z. et al. [45] also did not identify improvements in the composite severity score or in the need for admission to intensive care units after the nebulized administration of MgSO_4_. The absence of an effect on the severity score (SMD: –0.37; 95% CI: from –0.92 to 0.17) in our meta-analysis is consistent with the findings of Kumar, J. et al. [44] (SDM: –0,09; IC 95%: from –0,20 to +0,02), (SMD: –0.09; 95% CI: from –0.20 to +0.02), and could be explained by the high degree of variability in the measurement instruments used, the timing of the clinical response measurement (frequently <60 min post intervention), and the concomitant use of highly effective treatments, which reduce the ability to detect additional effects attributable to magnesium sulfate.

Regarding the length of hospital stay, no significant benefit was observed either (SMD: –0.75; 95% CI: from –1.90 to 0.40), a result that aligns with the reports of Mohammed et al. [20] and Su et al. [43], who also found no statistically significant reductions in this outcome. The high degree of heterogeneity observed in this analysis (I^2^ > 90%) limits the precision of the pooled estimate, which could be due to differences in the definition of “hospital discharge,” variations in hospitalization practices between centers and countries, and the absence of uniform clinical criteria to define clinical improvement or resolution. Regarding the route of administration, our subgroup analyses on various outcomes did not identify statistically significant differences between intravenous and nebulized magnesium sulfate. This finding partially contrasts with the reports of Shan, Z. et al. [23] and Su, Z. et al. [43], who observed significant beneficial effects, particularly with intravenous administration, in the pediatric population. A possible explanation for this discrepancy lies in the methodological design. In our meta-analysis, studies with both routes were included in the overall analysis and subsequently compared through subgroups, which allowed for the integration of a larger volume of evidence but also introduced a high degree of clinical heterogeneity (I^2^ > 90% in several outcomes), likely related to variations in dose, administration time, severity criteria, and population characteristics. In contrast, the studies that reported the superiority of the intravenous route conducted more specific analyses, exclusively evaluating children and excluding trials with disparate clinical features, which may have reduced variability and increased the power to detect differences. From a safety perspective, our findings are congruent with previous studies, such as those by Cheuk, D. et al. [42]; Kumar, J. et al. [44]; and Rowe, B. et al. [46], in which adverse events associated with magnesium sulfate were mild, transient, and not clinically significant. The consistency in this safety profile reinforces the feasibility of its use, especially in pediatric emergency departments, provided that protocolized doses and adequate supervision are used. On the other hand, the absence of information on mortality in the included studies coincides with what has been observed in previous reviews, where no randomized controlled trial evaluated this outcome as primary or secondary. Although the low mortality rate associated with asthmatic crises in childhood might justify this omission, its exclusion limits the comprehensive analysis of the impact of magnesium sulfate on vital prognosis and raises the need to incorporate this critical outcome in future well-designed clinical trials.

### 4.3. Mechanisms of Action of MgSO_4_ Supporting Its Utility in Asthmatic Crises

MgSO_4_ has been extensively investigated as an adjuvant therapy in the management of acute asthma, particularly in moderate or severe cases that show a poor response to first-line treatment (β_2_-adrenergics and corticosteroids) [47,48]. Its therapeutic action is based on various pathophysiological mechanisms, both classical and emerging, which intervene at the levels of the bronchial smooth muscle, the immunoinflammatory system, and neuroepithelial signaling [49,50].

The most consistent and clinically relevant mechanism of MgSO_4_ is the inhibition of calcium ion influx through voltage-dependent L-type channels located in bronchial smooth muscle cells [51,52]. By blocking these channels, magnesium prevents the intracellular increase of Ca^2+^, which is required for the activation of the calcium–calmodulin complex and, consequently, of myosin light-chain kinase, a critical enzyme in actin–myosin contraction [53]. This pathway culminates in the decreased phosphorylation of the myosin light chain and, therefore, in bronchial relaxation. This action occurs rapidly and synergistically with other molecules such as β_2_-adrenergic agonists [54].

On the other hand, it has been described that MgSO_4_ has a neuromodulatory effect, as it reduces the release of acetylcholine from parasympathetic presynaptic terminals by blocking P/Q-type calcium channels [55]. This limits the activation of M3 muscarinic receptors on bronchial smooth muscle, thus reducing the vagal bronchoconstrictor tone. This pathway is relevant in severe asthmatic crises, where an exacerbation of cholinergic activity, mediated by irritant reflexes and proinflammatory cytokines, has been documented [56].

From an immunological point of view, it has been demonstrated in animal models that magnesium stabilizes the mast cell membrane, decreasing degranulation induced by immunoglobulin E and the consequent releases of histamine, prostaglandin D2, and leukotrienes [57]. This action may be mediated by interference with phospholipase C and the inhibition of the protein kinase C pathway, which are essential routes in mast cell signaling. Furthermore, different studies have shown that Mg^2+^ can interfere with the activation of NF-κB, a central pathway in the transcription of proinflammatory genes, such as TNF-α, IL-4, IL-5, and IL-13, all of which are implicated in asthma pathophysiology [58].

Some research suggests that MgSO_4_ enhances nitric oxide (NO) signaling by promoting the activity of endothelial nitric oxide synthase, thereby elevating NO levels in the bronchial epithelium. This increase stimulates soluble guanylate cyclase, which, in turn, raises intracellular levels of cyclic guanosine monophosphate, a key second messenger that inhibits smooth muscle contractility by activating protein kinase G (PKG) and reducing the intracellular concentration of free calcium [59]. This pathway is synergistic with the classical cAMP-mediated pathway of β_2_-agonists, which would explain the additive effect documented in various clinical studies [60,61].

### 4.4. Limitations of the Included Studies

One of the methodological limitations recurrently identified in the included studies is the marked heterogeneity in intervention protocols. Differences in the route of administration of magnesium sulfate (nebulized versus intravenous), the doses used, the administration time, and the criteria for initiating treatment make the direct comparison of results difficult and limit the external validity of the findings. Likewise, considerable variability was observed in the scales used to assess the clinical severity of asthmatic crises, which adds an additional layer of inconsistency to the interpretation of the treatment’s efficacy. The lack of validated and comparable instruments for measuring this clinical outcome compromises the robustness of the pooled analyses and hinders the integration of results in systematic reviews. It is noteworthy that a significant number of studies (nine of the fourteen included) had an unclear risk of bias in various methodological domains, particularly concerning allocation concealment, the blinding of participants, or the outcome assessment. This variability in methodological quality requires a cautious interpretation of the results and highlights the need to improve the design and execution of future clinical trials. Finally, the absence of critical outcomes, such as mortality, should be mentioned. Although mortality in pediatric asthma is an infrequent event, its inclusion in larger-scale studies could provide relevant complementary information on the safety and overall impact of treatment with magnesium sulfate.

Another limitation evidenced is related to the poor uniformity in reporting key clinical variables that could act as effect modifiers, including the timing at which outcomes were evaluated in relation to the first-line medication administration. Given that the clinical effects of these drugs usually manifest between 3 and 4 h after their use, trials that measured clinical responses before that point could have overestimated the relative efficacy of MgSO_4_. However, most of the included studies did not clearly specify the interval between, for example, steroid administration and outcome evaluation, which prevented conducting a reliable subgroup analysis on this variable. This omission, together with variability in the use of co-interventions, limits the ability to precisely identify in which clinical context MgSO_4_ might be the most effective.

A relevant limitation of the present meta-analysis is the absence of an explicit diagnostic definition of asthma in most of the included studies. In general, the trials assumed that the participants already had a previous diagnosis, without detailing the clinical criteria used to establish it. This situation is especially relevant in the pediatric population, where the diagnosis of asthma, particularly in preschool-age children, can vary widely according to the clinical approach adopted. The lack of diagnostic uniformity may introduce biases in the selection of the studied population and limit comparability between studies. Although criteria were identified when reported, we recognize that this heterogeneity represents a methodological limitation that must be taken into account when interpreting the results of the analysis.

### 4.5. Limitations of the Review

Although pulmonary function parameters (such as FEV_1_ or PEF) are commonly used outcomes in asthma studies to evaluate bronchodilator responses, they were not included in our systematic review and meta-analysis protocol. This methodological decision was based on the clinical focus of the review, which was oriented toward more immediate and directly relevant outcomes in the context of acute exacerbations, such as the need for hospitalization, severity scores, and admission to intensive care units. We acknowledge that pulmonary function offers an objective assessment of the physiological response to treatment; however, its measurement in the pediatric population during acute episodes presents significant operational and clinical limitations, including a low degree of patient cooperation and the difficulty in obtaining reliable spirometry tests. Therefore, although its exclusion partially restricts the functional evaluation, it does not invalidate the robustness or applicability of the selected clinical outcomes, which better reflect the reality of management in emergency and hospital settings. It is possible that the inclusion of heterogeneous therapeutic regimens in the global analyses (with differences in doses, administration times, and indication criteria between the nebulized and intravenous routes) may have attenuated a possible differential effect between the two. This therapeutic variability could have diluted specific efficacy signals, which reinforces the need for future direct comparative studies that are well controlled and adequately stratified by route of administration, clinical severity, and other relevant covariates. While all the included studies focused on the pediatric population, it is important to note that this age group encompasses a wide range of physiological development, from infants to adolescents. This breadth implies possible divergences in the therapeutic responses to magnesium sulfate, given the differences in respiratory system maturation, pharmacokinetics, and bronchial reactivities. However, it was not possible to restrict the analysis to specific age subgroups due to the way the data were reported in the primary studies.

An important limitation of the findings of this review is the high degree of heterogeneity observed in some outcomes, particularly in clinical severity scores and durations of hospitalization. This variability can be explained by methodological differences between the included studies, such as the timing of the MgSO_4_ administration, the doses used, the baseline asthma severity, and the presence of co-interventions.

In addition to the above, there is the impossibility of performing subgroup analyses according to key clinical variables, such as the dose, timing of the administration, co-interventions, and baseline asthma severity, due to incomplete or heterogeneous reporting in the included studies. In several trials, the dose depended on the route of administration, and severity was not disaggregated, which limited the exploration of possible sources of heterogeneity.

### 4.6. Clinical Implications

The use of magnesium sulfate could represent a complementary strategy in the acute management of moderate-to-severe asthma in the pediatric population, particularly when the initial responses to conventional therapies are not optimal. Although some studies show a reduction in the hospitalization rate, this evidence should be interpreted with caution, given that it was not consistent with other outcomes. Furthermore, the lack of subgroup analyses according to the route of administration for some outcomes prevents establishing conclusions about their comparative efficacy.

In this context, magnesium sulfate can be considered as a second-line option, as suggested by some international guidelines, in selected clinical scenarios. Future research should focus on identifying clinical profiles that benefit the most from this intervention (such as patients with hypomagnesemia, non-eosinophilic asthma, or elevated cholinergic tone), as well as exploring its potential synergy with β_2_-agonists to optimize bronchodilator responses in specific contexts [62,63].

### 4.7. Recommendations for Future Research

The current evidence on the efficacy of magnesium sulfate as an adjuvant therapy in the treatment of moderate or severe asthmatic exacerbations in the pediatric population is promising but still has methodological limitations that prevent the establishment of definitive conclusions. In this context, the development of multicenter randomized clinical trials with a rigorous design, adequate sample sizes, and sufficient follow-up periods is considered as a priority to allow for the evaluation not only of the acute response but also of the subsequent clinical evolution and the risk of recurrence. Likewise, the adoption of validated and reproducible clinical scales to measure the severity of asthmatic crises in childhood is recommended, as many current investigations use heterogeneous or insufficiently described criteria. The incorporation of additional objective measures, such as respiratory functional parameters or inflammatory markers, could enrich the interpretation of the clinical effectiveness. On the other hand, it would be very useful for future studies to include direct comparisons between the different routes of administration, with a special emphasis on the safety, tolerability, and clinical efficacy profile of each. Although both routes have shown favorable results in terms of reducing hospitalization, the evidence is not conclusive regarding which one offers a greater benefit in specific populations.

## 5. Conclusions

This systematic review and meta-analysis demonstrates that in pediatric patients with moderate-to-severe exacerbated asthma, the use of magnesium sulfate is associated with a statistically significant reduction in the risk of hospitalization. However, no significant effects were found on other clinically relevant outcomes; this discrepancy requires a cautious interpretation of the results. These findings suggest that MgSO_4_ may be useful as a complementary intervention in the early stages of exacerbation management, but its impact on clinical progression and the need for intensive treatment remain uncertain. While the certainty of the evidence was generally low, this primarily reflects the methodological heterogeneity among the studies and does not invalidate the clinical potential of the drug but rather highlights the need for more standardized research with greater statistical power.

## Figures and Tables

**Table 1:** Characteristics of the studies included in the review.

Author	Year	Country	Study Type	Population	Inclusion Criteria	Exclusion Criteria	Outcomes
Asif, R. et al. [28]	2024	Pakistan	RCT	n = 68, I: 18, C: 50	(1) Children aged from 2 to 12 years, (2) presenting with a PRAM score of >4 and bronchial hyperreactivity	(1) Critically ill children requiring mechanical ventilation, (2) hypersensitivity or allergy to MgSO_4_	Severity score, length of hospital stay, and adverse events
Kadambari, A. et al. [29]	2023	India	RCT	n = 38, I: 19, C: 19	(1) Age between 1 and 12 years, (2) diagnosis of severe acute asthma, (3) non-responders to first-line treatment with salbutamol, ipratropium, and intravenous steroids.	(1) Coexisting diseases (pneumonia, tuberculosis, cystic fibrosis, and restrictive lung diseases), (2) renal or liver disease, (3) hypersensitivity to magnesium	Severity score and hospitalization.
Wongwaree, S. et al. [30]	2020	Thailand	RCT	n = 33, I: 16, C: 17	(1) Children aged from 2 to 15 years with moderate-to-severe asthma exacerbation and who did not improve after treatment with three doses of nebulized salbutamol, (2) patients with a PRAM score of ≥4.	(1) History of bronchopulmonary dysplasia, immunodeficiency, cystic fibrosis, primary ciliary dyskinesia, or chronic heart disease; (2) contraindication to using MgSO_4_ due to liver or renal disease; (3) allergy to magnesium, ipratropium bromide, or fenoterol	Severity score, length of hospital stay, and adverse effects
Kassisse, E. et al. [31]	2021	Venezuela	RCT	n = 131, I: 65, C: 66	(1) Patients between 2 and 12 years old with a severe acute asthma exacerbation, (2) patients with more than three asthma exacerbations that had improved with the use of bronchodilators in the emergency room	(1) Children with chronic respiratory, cardiac, renal, immunological, or hematological diseases; (2) patients hospitalized for asthma in the last 4 weeks or who had received IV MgSO_4_ in the last 2 weeks.	Severity score, hospitalization, ICU admission, and adverse effects
Schuh, S. et al. [32]	2020	Canada	RCT	n = 816, I: 409, C: 407	(1) Children aged from 2 to 17 years with a previous diagnosis of asthma, (2) previous episode of acute wheezing treated with an inhaled bronchodilator or systemic corticosteroid, (3) moderate or severe persistent asthma after an initial 1 h treatment period	(1) Children requiring immediate airway management, (2) received IV magnesium before study enrollment, (3) children with previously known hypersensitivity to magnesium	Severity score, hospitalization, and adverse effects
Turker, S. et al. [33]	2016	Turkey	RCT	n = 100, I: 50, C: 50	(1) Children between 3 and 15 years old with a diagnosis of asthma, under follow-up by an allergist; (2) admitted to the emergency room for a moderate asthma exacerbation	(1) Patients with associated chronic diseases, such as cystic fibrosis or bronchiectasis	Severity score and hospitalization
Alansarai, K. et al. [34]	2015	Qatar	RCT	n = 365, I: 191, C: 174	(1) Children aged from 2 to 14 years, previously known to have asthma; (2) presenting with a moderate or severe exacerbation, defined as a PRAM asthma severity score of ≥4	(1) Prematurity (34 weeks of gestation), (2) history of hypersensitivity to magnesium sulfate; history of neuromuscular, cardiac, or renal disease; (3) previous participation in the study or hemodynamic instability	Severity score, hospitalization, ICU admission, and adverse effects
Powell, C. et al. [35]	2013	United Kingdom	RCT	n = 476, I: 231, C: 245	(1) Patients between 2 and 16 years old, after 20 min of standard treatment without a response; (2) severe acute asthma exacerbation	NS	Severity score, hospitalization, length of hospital stay, ICU admission, and adverse events
Santana, J. et al. [36]	2001	Brazil	RCT	n = 33, I: 17, C: 16	(1) Children between 2 and 13 years old, (2) patients with severe acute asthma refractory to treatment	(1) Presence of other lung or heart diseases at hospital admission; (2) family history of supraventricular tachycardia, diabetes mellitus, or glucose intolerance; (3) delay in the administration of the study medications	Length of hospital stay and adverse effects.
Scarfone et al. [37]	2000	United States	RCT	n = 54, I: 24, C: 30	(1) Patients between 1 and 18 years old with a history of at least 1 episode of wheezing, with moderate-to-severe asthma exacerbation	(1) Patients who had used corticosteroids within the previous 72 h or had concurrent bronchiolitis, lobar pneumonia, croup, or suspected foreign body aspiration; (2) history of cystic fibrosis, bronchopulmonary dysplasia, congenital heart disease, or other chronic diseases	Severity score and adverse effects
Ciarallo, L. et al. [38]	2000	United States	RCT	n = 30, I: 16, C: 14	(1) Patients between 6 and 17.9 years old with an acute asthma exacerbation and who were admitted to the emergency department, (2) peak expiratory flow rate of less than 70%	(1) Body temperature greater than 38.5 °C, (2) use of theophylline within the week prior to the crisis, (3) history of renal or chronic lung disease other than asthma	Hospitalization and adverse effects
Gurkan, F. et al. [39]	1999	Turkey	RCT	n = 20, I: 10, C: 10	(1) Patients between 6 and 16 years old with an acute asthma exacerbation, (2) peak expiratory flow rate of less than 60%	NS	Severity score and adverse events
Devi, P. et al. [40]	1997	India	RCT	n = 47, I: 24, C: 23	(1) Children between 1 and 12 years old with a severe acute asthma exacerbation, (2) inadequate response to 3 initial doses of nebulized salbutamol at 20-minute intervals for 1 h	(1) Body temperature of greater than 38 °C, (2) systolic blood pressure below the 50th percentile for age	Length of hospital stay and adverse events
Ciarallo, L. et al. [41]	1996	United States	RCT	n = 31, I: 15, C: 16	(1) Patients aged from 6 to 18 years, with an acute asthma exacerbation	(1) Body temperature of greater than 38.5 °C, systolic blood pressure below the 25th percentile for age, (2) history of renal, pulmonary, or cardiac disease	Hospitalization and adverse effects

RCT, randomized controlled trial; n, total number of participants; I, intervention group; C, control group; PRAM, Pediatric Respiratory Assessment Measure; MgSO_4_, magnesium sulfate; IV, intravenous; ICU, intensive care unit; NS, not specified.

**Table 2:** Characteristics of the included population and the intervention.

Author, Year	Study Type	Population	Age	Male (%)	Asthma Exacerbation Severity	Route of Administration	Magnesium Sulfate Dose
Asif, R. et al., 2024 [28]	RCT	n = 68, I: 18, C: 50	From 2 to 12 years	46%	From moderate to severe	Nebulized	750 mg of MgSO_4_
Kadambari, A. et al., 2023 [29]	RCT	n = 38, I: 19, C: 19	From 1 to 12 years	45%	Severe	Nebulized	150 mg of MgSO_4_ in 2 mL of normal saline solution
Wongwaree, S. et al., 2020 [30]	RCT	n = 33, I: 16, C: 17	From 2 to 15 years	67%	Moderate	Nebulized	150 mg of MgSO_4_ in 2 mL of sterile water
Kassisse, E. et al., 2021 [31]	RCT	n = 131, I: 65, C: 66	From 2 to 12 years	55.7%	Severe	Intravenous	50 mg/kg of MgSO_4_ diluted in 30 cc of 5% dextrose solution
Schuh, S. et al., 2020 [32]	RCT	n = 816, I: 409, C: 407	From 2 to 17 years	63%	From moderate to severe	Nebulized	600 mg of MgSO_4_
Turker, S. et al., 2016 [33]	RCT	n = 100, I: 50, C: 50	3-15 years	54%	Moderate	Nebulized	150 mg of MgSO_4_ in 1.5 mL of normal saline solution
Alansarai, K. et al., 2015 [34]	RCT	n = 365, I: 191, C: 174	From 2 to 14 years	68%	From moderate to severe	Nebulized	800 mg of MgSO_4_ in 15 mL of normal saline solution
Powell, C. et al., 2013 [35]	RCT	n = 476, I: 231, C: 245	From 2 to 16 years	58%	Severe	Nebulized	150 mg of MgSO_4_ in 4 mL of normal saline solution
Santana, J. et al., 2001 [36]	RCT	n = 33, I: 17, C: 16	From 2 to 13 years	52%	Severe	Intravenous	50 mg/kg of MgSO_4_ diluted in saline solution
Scarfone et al., 2000 [37]	RCT	n = 54, I: 24, C: 30	From 1 to 18 years	52%	From moderate to severe	Intravenous	75 mg/kg of MgSO_4_ diluted in saline solution
Ciarallo, L. et al., 2000 [38]	RCT	n = 30, I: 16, C: 14	From 6 to 18 years	60%	From moderate to severe	Intravenous	25 mg/kg of MgSO_4_ diluted in 100 mL of saline solution
Gurkan, F. et al., 1999 [39]	RCT	n = 20, I: 10, C: 10	From 6 to 16 years	55%	From moderate to severe	Intravenous	40 mg/kg of MgSO_4_ diluted in 100 mL of saline solution
Devi, P. et al., 1997 [40]	RCT	n = 47, I: 24, C: 23	From 1 to 12 years	NR	Severe	Intravenous	100 mg/kg diluted in 30 mL of saline solution
Ciarallo, L. et al., 1996 [41]	RCT	n = 31, I: 15, C: 16	From 6 to 18 years	45.1%	From moderate to severe	Intravenous	25 mg/kg of MgSO_4_ diluted in 100 mL of saline solution

RCT, randomized controlled trial; n, total number of participants; I, intervention group; C, control group; NR, not reported; mg, milligram; mL, milliliter; cc, cubic centimeter; MgSO_4_, magnesium sulfate.

**Table 3:** Assessment of the quality of the evidence, using the Jadad scale.

Author(s)	Participants Were Assigned Using a Randomized Design	The Intervention Was Administered Under Double-Blind Conditions	Withdrawals and Losses to Follow-Up Were Described	The Randomization Method Was Adequately Reported	Selection Criteria are Clearly Described	Score
Alansarai, K. et al., 2015 [34]	1	1	1	1	1	5
Devi, P. et al., 1997 [40]	1	1	1	1	1	5
Asif, R. et al., 2024 [28]	1	0	1	0	1	3
Ciarallo, L. et al., 1996 [41]	1	1	1	1	1	5
Ciarallo, L. et al., 2000 [38]	1	1	1	1	1	5
Gurkan, F. et al., 1999 [39]	1	0	1	1	0	3
Kadambari, A. et al., 2023 [29]	1	0	0	1	1	3
Kassisse, E. et al., 2021 [31]	1	1	1	1	1	5
Powell, C. et al., 2013 [35]	1	1	1	1	0	4
Santana, J. et al., 2001 [36]	1	0	1	1	1	4
Scarfone, R. et al., 2000 [37]	1	0	1	1	1	4
Schuh, S. et al., 2020 [32]	1	1	1	1	1	5
Turker, S. et al., 2016 [33]	1	1	1	1	1	5
Wongwaree et al., 2022 [30]	1	1	1	1	1	5

**Table 4:** GRADE certainty of evidence for the outcomes.

Outcome	Effect Size (RR, MD or SMD)	Grade Certainty
Severity score	SDM: −0.37 (from −0.92 to 0.17)	⬤◯◯◯
Hospitalization	RR: 0.79 (from 0.67 to 0.94)	⬤⬤◯◯
Length of hospital stay	SDM: −0.75 (from −1.90 to 0.40)	⬤◯◯◯
Admission to ICU	RR: 0.62 (from 0.28 to 1.36)	⬤⬤⬤◯

⬤◯◯◯, Very low; ⬤⬤◯◯, Low; ⬤⬤⬤◯, Moderate.

## Data Availability

The raw data supporting the conclusions of this article are available in the published studies included in the meta-analysis and can be accessed through the respective publications.

## Supplementary Materials

The following supporting information can be downloaded at https://www.mdpi.com/article/10.3390/children12081064/s1: Figure S1: Subgroup analysis of magnesium sulfate administration timing and its effect on clinical severity scores. Figure S2: Subgroup analysis by presence of co-interventions and their effects on clinical severity scores. Figure S3: Subgroup analysis by method of magnesium sulfate administration and its effect on hospitalization outcomes. Figure S4: Subgroup analysis by presence of co-interventions and their effects on hospitalization outcomes Figure S5: Sensitivity analysis assessing the effect of magnesium sulfate on the asthma severity score after excluding the study by Asif, R. et al. [26]. Figure S6: Sensitivity analysis of the effect of magnesium sulfate on asthma severity scores, excluding non-double-blind studies. Figure S7: Sensitivity analysis of the effect of magnesium sulfate on hospitalization outcomes, excluding non-double-blind studies. Figure S8: Sensitivity analysis of the effect of magnesium sulfate on the length of hospital stay, excluding non-double-blind studies. Table S1: Therapeutic context of magnesium sulfate administration in the included studies. Table S2: Summary of findings (SoF) table detailing the certainty of evidence for each primary outcome included in the meta-analysis. Table S3: PRISMA checklist used to ensure transparency and completeness in the reporting of this systematic review and meta-analysis.
