# Supplementary material for: Effectiveness of Intravenous and Nebulized MgSO4 in Children with Asthma Exacerbation: A Systematic Review and Meta-Analysis of Clinical Trials
Source: Children (Basel). 2025 Aug 13;12(8):1064. doi: 10.3390/children12081064 (PMC12384798; doi:10.3390/children12081064)
Supplement: Supplementary file 1 [file children-12-01064-s001.zip › Table S1.pdf]

Table S1. Therapeutic context of magnesium sulfate administration in the included studies

Author, year	Study type	Population	Timing of $MgSO_4$ administratition	Prior intervention	Time from initial therapy to intervention	Co-intervention	Timing of Outcome Assessment
Asif R et al, 2024[28]	RCT	n=68, I:18, C:50	From the beginning	N/A	N/A	Nebulized SABA	NUR
Kadambari A et al, 2023 [29]	RCT	n=38, I:19, C:19	After standard initial management	I.V. Hydrocortisone	Not described	Nebulized SABA	NUR
Wongwaree S et al, 2020[30]	RCT	n=33, I:16, C:17	After standard initial management	I.V. Hydrocortisone	Not described	I.V. Hydrocortisone	NUR
Kassisse E et al, 2021 [31]	RCT	n=131, I:65, C:66	After standard initial management	SABA + I.V. hydrocortisone	Not described	None	NUR
Schuh S et al, 2020 [32]	RCT	n=816, I:409, C:407	After standard initial management	Oral prednisolone + SABA + SAMA	1 hour	Nebulized SABA	NUR
Turker S et al, 2016[33]	RCT	n=100, I:50, C:50	From the beginning	N/A	N/A	Nebulized SABA + I.V. methylprednisolone	NUR
Alansarai K et al, 2015[34]	RCT	n=365, I:191, C:174	After standard initial management	SABA + SAMA + I.V. methylprednisolone	1 hour	Nebulized SABA + SAMA + I.V. methylprednisolone	NUR
Powell C et al, 2013[35]	RCT	n=476, I:231, C:245	After standard initial management	Not described	< 1 hour	Nebulized SABA + SAMA	NUR
Santana J et al, 2001[36]	RCT	n=33, I:17, C:16	After standard initial management	SABA + I.V. hydrocortisone	Not described	None	NUR
Scarfone et al, 2000[37]	RCT	n=54, I:24, C:30	After standard initial management	SABA + I.V. methylprednisolone	Not described	Nebulized SABA + I.V. methylprednisolone	NUR
Ciarallo L et al, 2000[38]	RCT	n=30, I:16, C:14	After standard initial management	I.V. Methylprednisolone	Not described	None	NUR
Gurkan F et al, 1999[39]	RCT	n=20, I:10, C:10	After standard initial management	SABA + I.V. methylprednisolone	Not described	None	NUR
Devi P et al, 1997[40]	RCT	n=47, I:24, C:23	After standard initial management	SABA + I.V. hydrocortisone	1 hour	Nebulized SABA + I.V. hydrocortisone	NUR
Ciarallo L et al., 1996 [41]	RCT	n=31, I:15, C:16	After standard initial management	I.V. Methylprednisolone	Not described	Nebulized SABA	NUR

NUR: Not uniformly reported; N/A: Not aplicable; IV: intravenous; SABA: Short-Acting Beta-2 Agonist; SAMA: Short Acting Muscarinic Antagonist
