# Supplementary material for: Effectiveness of Intravenous and Nebulized MgSO4 in Children with Asthma Exacerbation: A Systematic Review and Meta-Analysis of Clinical Trials
Source: Children (Basel). 2025 Aug 13;12(8):1064. doi: 10.3390/children12081064 (PMC12384798; doi:10.3390/children12081064)
Supplement: Supplementary file 1 [file children-12-01064-s001.zip › Table S2 Summary of Findings (SoF) table detailing the certainty of evidence for each primary outcome included in the meta-analysis. .pdf]

Author(s): Víctor Hugo Estupiñán Pérez , Freiser Eceomo Cruz Mosquera1, Mayerli de la Rosa Caldas, Olmer Alexander Pantoja Rodríguez, and Yamil Liscano1.
Question: $MgSO_4$ compared to placebo or standard care in Children with Asthma Exacerbation
Setting:
Bibliography:

Certainty assessment							№ of patients		Effect		Certainty	Importance
№ of studies	Study design	Risk of bias	Inconsistency	Indirectness	Imprecision	Other considerations	$MgSO_4$	placebo or standard care	Relative (95% CI)	Absolute (95% CI)		
**Asthma exacerbation severity score**												
10	randomised trials	serious[a]	serious[b]	not serious	serious[c]	none	1030	1067	-	**0** (0 to 0)	⊕◯◯◯ Very low[a,b,c]	
**Hospitalization**												
8	randomised trials	serious[d]	serious[e]	not serious	not serious	none	471/996 (47.3%)	547/991 (55.2%)	**RR 0.79** (0.67 to 0.94)	**116 fewer per 1,000** (from 182 fewer to 33 fewer)	⊕⊕◯◯ Low[d,e]	
**Length of Hospital Stay**												
5	randomised trials	serious[f]	serious[g]	not serious	serious[h]	none	303	350	-	**0** (0 to 0)	⊕◯◯◯ Very low[f,g,h]	
**ICU admission**												
3	randomised trials	not serious	not serious	not serious	serious[i]	none	9/471 (1.9%)	16/478 (3.3%)	**RR 0.62** (0.28 to 1.36)	**13 fewer per 1,000** (from 24 fewer to 12 more)	⊕⊕⊕◯ Moderate[i]	

CI: confidence interval; **RR:** risk ratio

Explanations

a. The certainty of evidence was downgraded by one level due to risk of bias. Although most included studies show a low risk of bias across several domains, a substantial proportion of studies presented an unclear risk of bias in critical areas such as blinding of participants and personnel. Additionally, one study demonstrated a high risk of bias in this domain. These concerns suggest a moderate likelihood of systematic bias that could potentially affect the overall reliability of the findings.
b. The certainty of evidence was downgraded by one level due to inconsistency. There is substantial statistical heterogeneity among the included studies, with an I^2 value of 90%, indicating considerable variability in effect estimates. This high level of heterogeneity suggests differences in study populations, interventions, or methodologies that may reduce confidence in the consistency and applicability of the pooled results.
c. The certainty of evidence was downgraded by one level due to imprecision. The 95% confidence interval for the overall effect estimate ranges from -0.92 to 0.17, crossing the line of no effect and encompassing both meaningful benefit and the possibility of no effect or harm. Additionally, the result was not statistically significant ($p = 0.16$), indicating uncertainty about the true effect size. This limits the confidence in the estimate of effect.
d. The certainty of evidence was downgraded by one level due to risk of bias. Among the eight included studies, three had a low risk of bias across all domains. However, four studies presented unclear risk in several key domains, particularly in allocation concealment and blinding. One study (Kadambari et al., 2023) had a high risk of bias in participant and personnel blinding. These issues raise moderate concerns about potential systematic bias affecting the reliability of the effect estimates.
e. The certainty of evidence was downgraded by one level due to inconsistency. There was substantial statistical heterogeneity among the included studies, with an I^2 of 69% and a significant Chi^2 test ($p = 0.002$), suggesting true variability beyond chance. The variation in effect sizes across studies, with confidence intervals that do not fully overlap, indicates inconsistency in the magnitude and possibly the direction of effects, reducing confidence in the pooled estimate.
f. Several included studies (Devi et al., Santana et al., Asif et al.) had unclear risk of bias in key domains, particularly regarding allocation concealment and blinding of participants and personnel. Only two studies (Powell et al., Wongwaree et al.) had low risk of bias across all domains. These concerns lower confidence in the internal validity of the findings.
g. There was considerable heterogeneity among studies ($I^2 = 91\%$), with large variability in effect sizes and limited overlap of confidence intervals. These discrepancies suggest true differences in effects likely due to clinical or methodological diversity, reducing confidence in the consistency of the results.
h. The 95% confidence interval of the pooled estimate (SMD: -0.75; 95% CI: -1.90 to 0.40) crosses the line of no effect and includes both substantial benefit and no effect. The result is not statistically significant ($p = 0.14$), and the wide interval reflects uncertainty in the true magnitude and direction of the effect.
i. The 95% confidence interval is wide and includes both a substantial reduction and potential increase in risk (RR: 0.62 [0.28 to 1.36]). The total number of events is low ($n = 25$), which limits the precision and stability of the effect estimate.
