# Supplementary material for: Effectiveness of Intravenous and Nebulized MgSO4 in Children with Asthma Exacerbation: A Systematic Review and Meta-Analysis of Clinical Trials
Source: Children (Basel). 2025 Aug 13;12(8):1064. doi: 10.3390/children12081064 (PMC12384798; doi:10.3390/children12081064)
Supplement: Supplementary file 1 [file children-12-01064-s001.zip › Figure S1-S8.pdf]

SUPPLEMENTARY MATERIAL

Figure S1. Subgroup analysis of magnesium sulfate administration timing and its effect on clinical severity scores

Study or Subgroup	Experimental Mean	Experimental SD	Experimental Total	Control Mean	Control SD	Control Total	Weight	Std. mean difference IV, Random, 95% CI
**Initial treatment**								
Asif R et al. 2024	0.32	0.43	18	0.68	0.65	50	9.6%	-0.59 [-1.14 , -0.04]
Tuker S et al. 2016	2.06	1.4	50	1.68	1.8	50	11.0%	0.23 [-0.16 , 0.63]
**Subtotal (HKSJ[a])**			**68**			**100**	**20.5%**	**-0.16 [-5.40 , 5.08]**
Test for overall effect: T = 0.38, df = 1 (P = 0.77)								
Heterogeneity: Tau² (DL[b]) = 0.28; Chi² = 5.75, df = 1 (P = 0.02); I² = 83%								
**Post SABA/SAMA/CS**								
Alansarai K et al. 2015	3.9	1.6	191	3.3	1.5	174	12.4%	0.39 [0.18 , 0.59]
Gurkan F et al. 1999	4	0.5	10	5.5	0.5	10	4.2%	-2.87 [-4.20 , -1.55]
Kadambari et al. 2023	6.95	1.29	19	7.63	1.03	19	8.6%	-0.57 [-1.22 , 0.08]
Kassisse E et al. 2021	4.9	2.5	65	8.6	2.8	66	11.1%	-1.39 [-1.77 , -1.00]
Powell C. et al 2013	4.72	1.37	228	4.95	1.4	244	12.5%	-0.17 [-0.35 , 0.02]
Scarfone R et al. 2000	5.9	2.5	24	6.8	2.6	30	9.6%	-0.35 [-0.89 , 0.19]
Schuch S et al 2020	3.84	1.94	409	4.13	2.04	407	12.7%	-0.15 [-0.28 , -0.01]
Wongwaree S et al. 2022	1.38	1.4	16	1.29	1.7	17	8.3%	0.06 [-0.63 , 0.74]
**Subtotal (HKSJ[a])**			**962**			**967**	**79.5%**	**-0.44 [-1.13 , 0.26]**
Test for overall effect: T = 1.49, df = 7 (P = 0.18)								
Heterogeneity: Tau² (DL[b]) = 0.23; Chi² = 84.39, df = 7 (P < 0.00001); I² = 92%								
**Total (HKSJ[a])**			**1030**			**1067**	**100.0%**	**-0.37 [-0.92 , 0.17]**

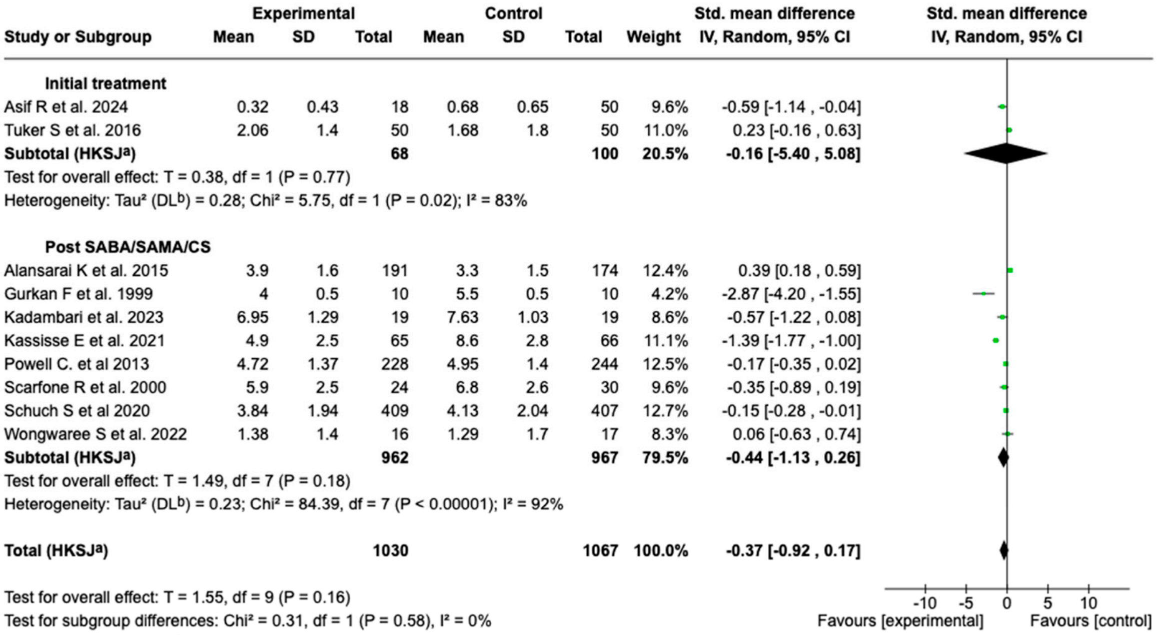

Test for overall effect: T = 1.55, df = 9 (P = 0.16)
Test for subgroup differences: Chi² = 0.31, df = 1 (P = 0.58), I² = 0%
Heterogeneity: Tau² (DL[b]) = 0.22; Chi² = 90.46, df = 9 (P < 0.00001); I² = 90%

Footnotes
[a]CI calculated by Hartung-Knapp-Sidik-Jonkman method.
[b]Tau² calculated by DerSimonian and Laird method.

Figure S2. Subgroup analysis by presence of co-interventions and its effect on clinical severity scores

Study or Subgroup	Experimental Mean	Experimental SD	Experimental Total	Control Mean	Control SD	Control Total	Weight	Std. mean difference IV, Random, 95% CI
**Co-intervention**								
Alansarai K et al. 2015	3.9	1.6	191	3.3	1.5	174	12.4%	0.39 [0.18 , 0.59]
Asif R et al. 2024	0.32	0.43	18	0.68	0.65	50	9.6%	-0.59 [-1.14 , -0.04]
Kadambari et al. 2023	6.95	1.29	19	7.63	1.03	19	8.6%	-0.57 [-1.22 , 0.08]
Powell C. et al 2013	4.72	1.37	228	4.95	1.4	244	12.5%	-0.17 [-0.35 , 0.02]
Scarfone R et al. 2000	5.9	2.5	24	6.8	2.6	30	9.6%	-0.35 [-0.89 , 0.19]
Schuch S et al 2020	3.84	1.94	409	4.13	2.04	407	12.7%	-0.15 [-0.28 , -0.01]
Tuker S et al. 2016	2.06	1.4	50	1.68	1.8	50	11.0%	0.23 [-0.16 , 0.63]
Wongwaree S et al. 2022	1.38	1.4	16	1.29	1.7	17	8.3%	0.06 [-0.63 , 0.74]
**Subtotal (HKSJ[a])**			**955**			**991**	**84.8%**	**-0.09 [-0.37 , 0.20]**
Test for overall effect: T = 0.73, df = 7 (P = 0.49)								
Heterogeneity: Tau² (DL[b]) = 0.07; Chi² = 29.84, df = 7 (P = 0.0001); I² = 77%								
**Without co-intervention**								
Gurkan F et al. 1999	4	0.5	10	5.5	0.5	10	4.2%	-2.87 [-4.20 , -1.55]
Kassisse E et al. 2021	4.9	2.5	65	8.6	2.8	66	11.1%	-1.39 [-1.77 , -1.00]
**Subtotal (HKSJ[a])**			**75**			**76**	**15.2%**	**-1.99 [-11.27 , 7.29]**
Test for overall effect: T = 2.72, df = 1 (P = 0.22)								
Heterogeneity: Tau² (DL[b]) = 0.86; Chi² = 4.47, df = 1 (P = 0.03); I² = 78%								
**Total (HKSJ[a])**			**1030**			**1067**	**100.0%**	**-0.37 [-0.92 , 0.17]**

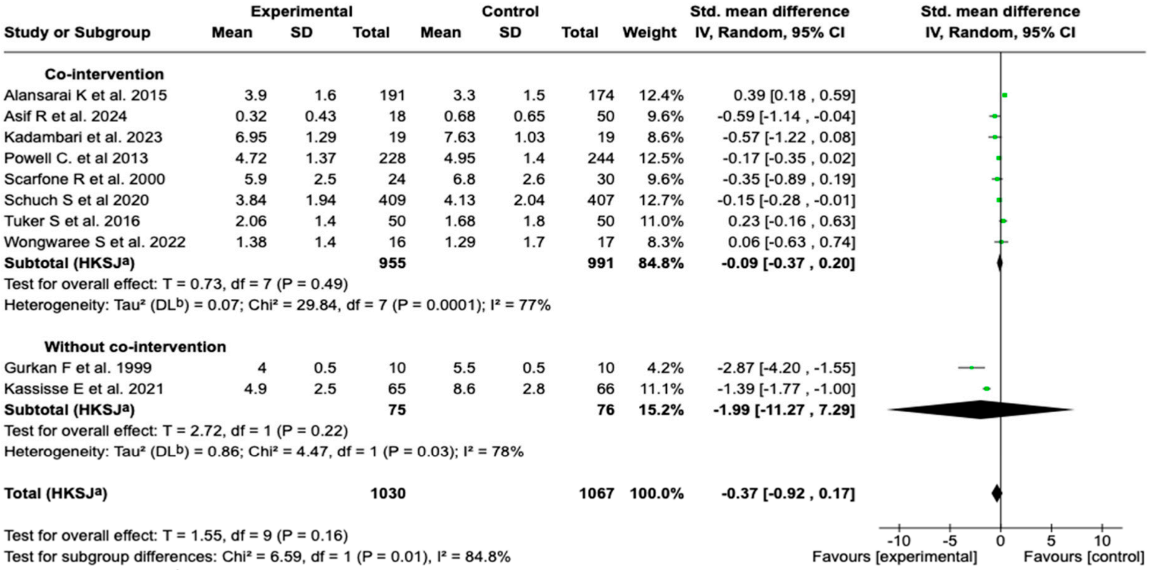

Test for overall effect: T = 1.55, df = 9 (P = 0.16)
Test for subgroup differences: Chi² = 6.59, df = 1 (P = 0.01), I² = 84.8%
Heterogeneity: Tau² (DL[b]) = 0.22; Chi² = 90.46, df = 9 (P < 0.00001); I² = 90%

Footnotes
[a]CI calculated by Hartung-Knapp-Sidik-Jonkman method.
[b]Tau² calculated by DerSimonian and Laird method.

Figure S3. Subgroup analysis by method of magnesium sulfate administration and its effect on hospitalization outcomes.

Study or Subgroup	Experimental Events	Experimental Total	Control Events	Control Total	Weight	Risk ratio IV, Random, 95% CI
**Nebulized**						
Alansarai K et al. 2015	1	191	1	174	0.3%	0.91 [0.06 , 14.45]
Kadambari et al. 2023	10	19	15	19	7.7%	0.67 [0.41 , 1.08]
Powell C. et al 2013	215	231	238	245	27.0%	0.96 [0.92 , 1.00]
Schuch S et al 2020	178	409	194	407	22.1%	0.91 [0.79 , 1.06]
Tuker S et al. 2016	6	50	6	50	2.1%	1.00 [0.35 , 2.89]
**Subtotal (HKSJ[a])**		**900**		**895**	**59.2%**	**0.95 [0.91 , 1.00]**
Total events:	410		454			
Test for overall effect: T = 3.06, df = 4 (P = 0.04)						
Heterogeneity: Tau² (DL[b]) = 0.00; Chi² = 2.47, df = 4 (P = 0.65); I² = 0%						
**Intravenous**						
Ciarallo L et al. 1996	11	15	16	16	13.1%	0.74 [0.54 , 1.02]
Ciarallo L et al. 2000	8	16	14	14	7.8%	0.52 [0.32 , 0.84]
Kassisse E et al. 2021	42	65	63	66	19.9%	0.68 [0.56 , 0.82]
**Subtotal (HKSJ[a])**		**96**		**96**	**40.8%**	**0.67 [0.50 , 0.90]**
Total events:	61		93			
Test for overall effect: T = 5.88, df = 2 (P = 0.03)						
Heterogeneity: Tau² (DL[b]) = 0.00; Chi² = 1.49, df = 2 (P = 0.48); I² = 0%						
**Total (HKSJ[a])**		**996**		**991**	**100.0%**	**0.79 [0.67 , 0.94]**
Total events:	471		547			
Test for overall effect: T = 3.17, df = 7 (P = 0.02)						
Test for subgroup differences: Chi² = 25.25, df = 1 (P < 0.00001), I² = 96.0%						
Heterogeneity: Tau² (DL[b]) = 0.02; Chi² = 22.51, df = 7 (P = 0.002); I² = 69%						

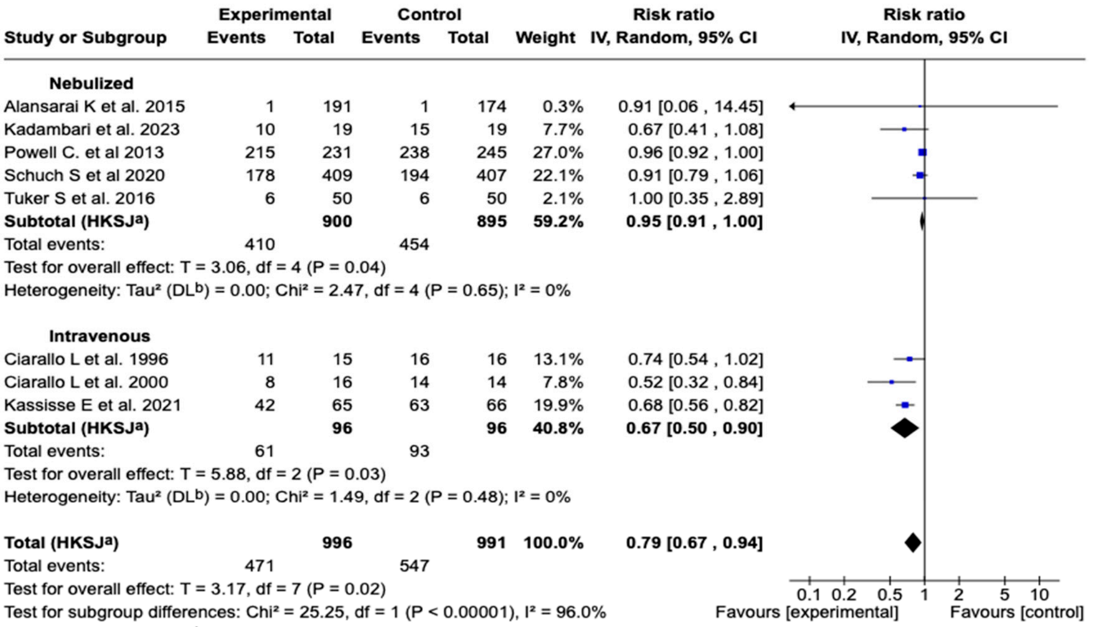

Footnotes

[a]CI calculated by Hartung-Knapp-Sidik-Jonkman method.

[b]Tau² calculated by DerSimonian and Laird method.

Figure S4. Figure S4. Subgroup analysis by presence of co-interventions and its effect on hospitalization outcomes.

Study or Subgroup	Experimental Events	Experimental Total	Control Events	Control Total	Weight	Risk ratio M-H, Fixed, 95% CI
**Co-intervention**						
Alansarai K et al. 2015	1	191	1	174	0.2%	0.91 [0.06 , 14.45]
Ciarallo L et al. 1996	11	15	16	16	3.0%	0.74 [0.54 , 1.02]
Kadambari et al. 2023	10	19	15	19	2.8%	0.67 [0.41 , 1.08]
Powell C. et al 2013	215	231	238	245	42.7%	0.96 [0.92 , 1.00]
Schuch S et al 2020	178	409	194	407	35.9%	0.91 [0.79 , 1.06]
Tuker S et al. 2016	6	50	6	50	1.1%	1.00 [0.35 , 2.89]
**Subtotal**		**915**		**911**	**85.6%**	**0.92 [0.86 , 0.99]**
Total events:	421		470			
Test for overall effect: Z = 2.26 (P = 0.02)						
Heterogeneity: Chi² = 6.81, df = 5 (P = 0.23); I² = 27%						
**Without co-intervention**						
Ciarallo L et al. 2000	8	16	14	14	2.8%	0.52 [0.32 , 0.84]
Kassisse E et al. 2021	42	65	63	66	11.5%	0.68 [0.56 , 0.82]
**Subtotal**		**81**		**80**	**14.4%**	**0.65 [0.54 , 0.77]**
Total events:	50		77			
Test for overall effect: Z = 4.87 (P < 0.00001)						
Heterogeneity: Chi² = 1.05, df = 1 (P = 0.31); I² = 5%						
**Total**		**996**		**991**	**100.0%**	**0.88 [0.83 , 0.94]**
Total events:	471		547			
Test for overall effect: Z = 3.77 (P = 0.0002)						
Test for subgroup differences: Chi² = 13.65, df = 1 (P = 0.0002), I² = 92.7%						
Heterogeneity: Chi² = 30.25, df = 7 (P < 0.0001); I² = 77%						

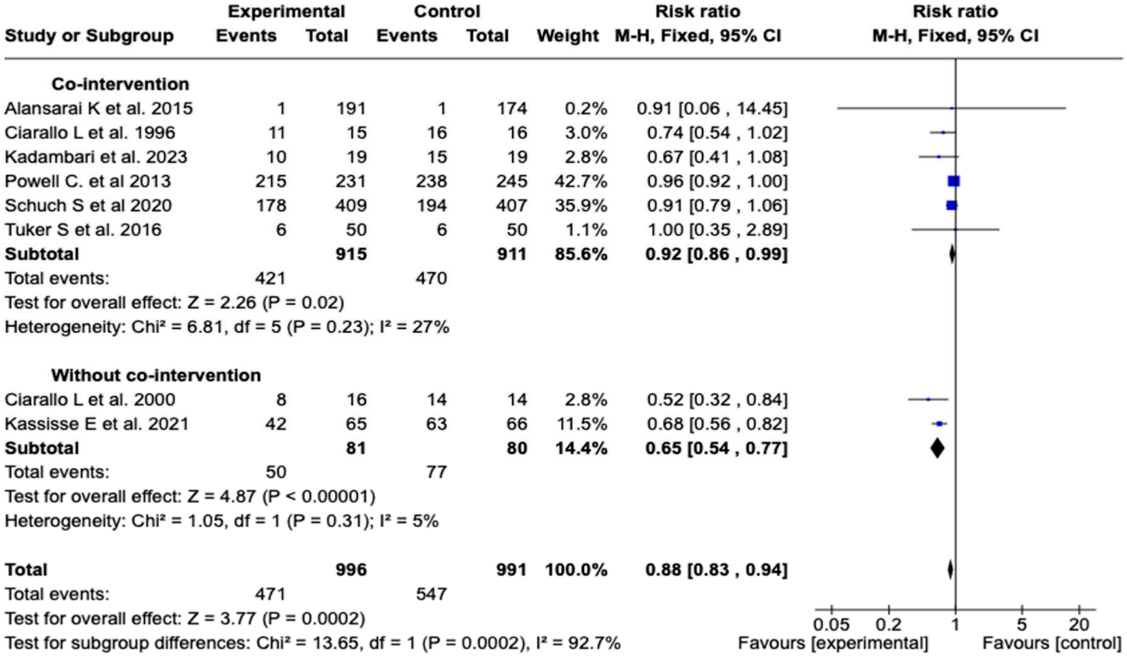

Figure S5. Sensitivity analysis assessing the effect of magnesium sulfate on asthma severity score after excluding the study by Asif R et al. [28]

Study or Subgroup	Experimental Mean	Experimental SD	Experimental Total	Control Mean	Control SD	Control Total	Weight	Std. mean difference IV, Random, 95% CI
✓ Alansarai K et al. 2015	3.9	1.6	191	3.3	1.5	174	13.7%	0.39 [0.18 , 0.59]
✕ Asif R et al. 2024	0.32	0.43	18	0.68	0.65	50	0.0%	-0.59 [-1.14 , -0.04]
✓ Gurkan F et al. 1999	4	0.5	10	5.5	0.5	10	4.7%	-2.87 [-4.20 , -1.55]
✓ Kadambari et al. 2023	6.95	1.29	19	7.63	1.03	19	9.6%	-0.57 [-1.22 , 0.08]
✓ Kassisse E et al. 2021	4.9	2.5	65	8.6	2.8	66	12.2%	-1.39 [-1.77 , -1.00]
✓ Powell C. et al 2013	4.72	1.37	228	4.95	1.4	244	13.8%	-0.17 [-0.35 , 0.02]
✓ Scarfone R et al. 2000	5.9	2.5	24	6.8	2.6	30	10.7%	-0.35 [-0.89 , 0.19]
✓ Schuch S et al 2020	3.84	1.94	409	4.13	2.04	407	14.0%	-0.15 [-0.28 , -0.01]
✓ Tuker S et al. 2016	2.06	1.4	50	1.68	1.8	50	12.1%	0.23 [-0.16 , 0.63]
✓ Wongwaree S et al. 2022	1.38	1.4	16	1.29	1.7	17	9.2%	0.06 [-0.63 , 0.74]
**Total (HKSJ[a])**			**1012**			**1017**	**100.0%**	**-0.35 [-0.97 , 0.27]**

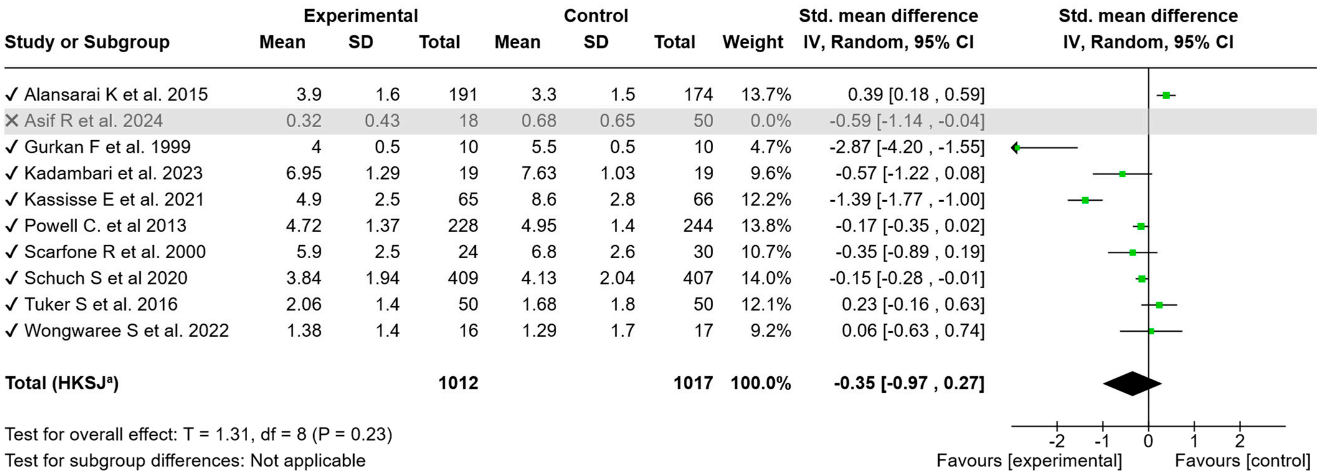

Test for overall effect: T = 1.31, df = 8 (P = 0.23)
Test for subgroup differences: Not applicable
Heterogeneity: Tau² (DL[b]) = 0.22; Chi² = 87.72, df = 8 (P < 0.00001); I² = 91%

Footnotes
[a]CI calculated by Hartung-Knapp-Sidik-Jonkman method.
[b]Tau² calculated by DerSimonian and Laird method.

Figure S6. Sensitivity analysis of the effect of magnesium sulfate on asthma severity scores, excluding non–double-blind studies.

✔ Alansarai K et al. 2015	3.9	1.6	191	3.3	1.5	174	18.4%	0.39 [0.18 , 0.59]
✖ Asif R et al. 2024	0.32	0.43	18	0.68	0.65	50	0.0%	-0.59 [-1.14 , -0.04]
✖ Gurkan F et al. 1999	4	0.5	10	5.5	0.5	10	0.0%	-2.87 [-4.20 , -1.55]
✖ Kadambari et al. 2023	6.95	1.29	19	7.63	1.03	19	0.0%	-0.57 [-1.22 , 0.08]
✔ Kassisse E et al. 2021	4.9	2.5	65	8.6	2.8	66	16.2%	-1.39 [-1.77 , -1.00]
✔ Powell C. et al 2013	4.72	1.37	228	4.95	1.4	244	18.6%	-0.17 [-0.35 , 0.02]
✖ Scarfone R et al. 2000	5.9	2.5	24	6.8	2.6	30	0.0%	-0.35 [-0.89 , 0.19]
✔ Schuch S et al 2020	3.84	1.94	409	4.13	2.04	407	18.9%	-0.15 [-0.28 , -0.01]
✔ Tuker S et al. 2016	2.06	1.4	50	1.68	1.8	50	16.0%	0.23 [-0.16 , 0.63]
✔ Wongwaree S et al. 2022	1.38	1.4	16	1.29	1.7	17	11.9%	0.06 [-0.63 , 0.74]
**Total (HKSJ[a])**			**959**			**958**	**100.0%**	**-0.17 [-0.83 , 0.49]**

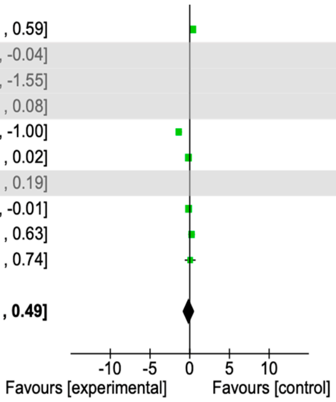

Test for overall effect: T = 0.65, df = 5 (P = 0.54)

Heterogeneity: Tau² (DL[b]) = 0.19; Chi² = 68.33, df = 5 (P < 0.00001); I² = 93%

Footnotes
[a]CI calculated by Hartung-Knapp-Sidik-Jonkman method.
[b]Tau² calculated by DerSimonian and Laird method.

Figure S7. Sensitivity analysis of the effect of magnesium sulfate on hospitalization outcomes, excluding non–double-blind studies.

Study or Subgroup	Experimental Events	Experimental Total	Control Events	Control Total	Weight	Risk ratio IV, Random, 95% CI
✓ Alansarai K et al. 2015	1	191	1	174	0.4%	0.91 [0.06 , 14.45]
✓ Ciarallo L et al. 1996	11	15	16	16	14.2%	0.74 [0.54 , 1.02]
✓ Ciarallo L et al. 2000	8	16	14	14	8.4%	0.52 [0.32 , 0.84]
✕ Kadambari et al. 2023	10	19	15	19	0.0%	0.67 [0.41 , 1.08]
✓ Kassisse E et al. 2021	42	65	63	66	21.6%	0.68 [0.56 , 0.82]
✓ Powell C. et al 2013	215	231	238	245	29.3%	0.96 [0.92 , 1.00]
✓ Schuch S et al 2020	178	409	194	407	23.9%	0.91 [0.79 , 1.06]
✓ Tuker S et al. 2016	6	50	6	50	2.2%	1.00 [0.35 , 2.89]
**Total (HKSJ[a])**		**977**		**972**	**100.0%**	**0.81 [0.66 , 0.98]**
Total events:	461		532			

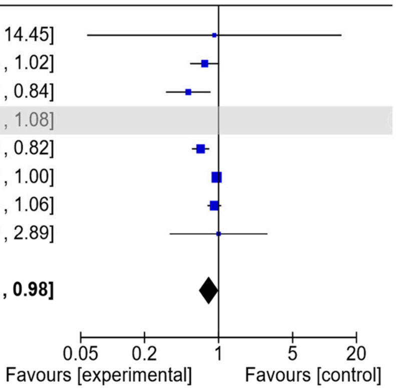

Test for overall effect: T = 2.74, df = 6 (P = 0.03)

Heterogeneity: Tau² (DL[b]) = 0.02; Chi² = 20.67, df = 6 (P = 0.002); I² = 71%

Footnotes

[a]CI calculated by Hartung-Knapp-Sidik-Jonkman method.

[b]Tau² calculated by DerSimonian and Laird method.

Figure S8. Sensitivity analysis of the effect of magnesium sulfate on length of hospital stay, excluding non–double-blind studies.

Study or Subgroup	Experimental Mean	Experimental SD	Experimental Total	Control Mean	Control SD	Control Total	Weight	Std. mean difference IV, Random, 95% CI
✓ Asif R et al. 2024	2	0.77	18	4.06	1.56	50	24.9%	-1.45 [-2.05 , -0.86]
✓ Devi P et al. 1997	3.6	6.8	24	18.9	7.7	23	23.8%	-2.07 [-2.79 , -1.35]
✓ Powell C. et al 2013	26.3	6.9	228	27.1	7.2	244	27.3%	-0.11 [-0.29 , 0.07]
✕ Santana J et al. 2001	6.12	2.47	17	6.56	3.67	16	0.0%	-0.14 [-0.82 , 0.55]
✓ Wongwaree S et al. 2022	3.76	1.1	16	3.86	1.5	17	24.1%	-0.07 [-0.76 , 0.61]
**Total (HKSJ[a])**			**286**			**334**	**100.0%**	**-0.90 [-2.48 , 0.67]**

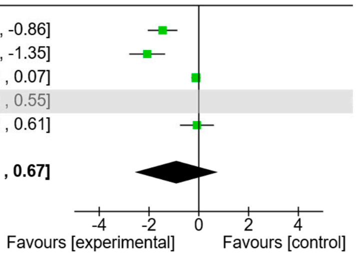

Test for overall effect: T = 1.82, df = 3 (P = 0.17)

Heterogeneity: Tau² (DL[b]) = 0.85; Chi² = 42.28, df = 3 (P < 0.00001); I² = 93%

Footnotes

[a]CI calculated by Hartung-Knapp-Sidik-Jonkman method.

[b]Tau² calculated by DerSimonian and Laird method.
